# Association of the atherogenic index of plasma and high-sensitivity C-reactive protein with incident cardiovascular disease: evidence from a national cohort of middle-aged and older Chinese adults

**DOI:** 10.3389/fendo.2025.1618157

**Published:** 2025-08-06

**Authors:** Ping-an Lian, Fei Xie, Wei Zhang, Shuai Cheng, Yun-fei Zhao, Lin Li, Shan-fu Liang, Zhan-zhan Zhu, Jun-yue Zhang, Jiao-jiao Cui, Jie Du, Lei Yin, Shen-wei Zhang

**Affiliations:** ^1^ Department of Cardiology, Seventh People’s Hospital of Zhengzhou, Zhengzhou, Henan, China; ^2^ Biotherapy Institute, Henan Academy of Innovations in Medical Science, Zhengzhou, Henan, China; ^3^ Department of Ophthalmology, Seventh People’s Hospital of Zhengzhou, Zhengzhou, Henan, China

**Keywords:** atherogenic index of plasma, C-reactive protein, cardiovascular disease, inflammation, longitudinal cohort study, CHARLS

## Abstract

**Background:**

The atherogenic index of plasma (AIP), an emerging biomarker of lipid dysregulation, and high-sensitivity C-reactive protein (hs-CRP), an established marker of inflammation, are both implicated in the development of cardiovascular disease (CVD). However, their joint impact on CVD risk and the underlying mediation mechanisms remain unclear.

**Methods:**

This study used data from the China Health and Retirement Longitudinal Study (CHARLS), including 8,763 adults aged ≥45 years with up to 9 years of follow-up. Baseline AIP and hs-CRP levels were measured, and participants were divided into four groups based on the AIP median and hs-CRP threshold (1 mg/L). Multivariable Cox models assessed associations with CVD. Mediation analyses examined direct and indirect effects, including bidirectional mediation. Additive interaction was evaluated using the relative excess risk due to interaction (RERI), and predictive performance was assessed via Receiver Operating Characteristic Curve (ROC) curve analysis.

**Results:**

A total of 1,693 participants developed CVD during the follow-up period. Higher levels of AIP and hs-CRP were independently associated with CVD. Joint analysis showed that, compared with individuals with AIP below the median and hs-CRP <1 mg/L, those with elevated levels of both AIP and hs-CRP had the highest risks of CVD (HR: 1.655; 95% CI: 1.455-1.883), heart disease (HR: 1.402; 95% CI: 1.207-1.628), and stroke (HR: 2.207; 95% CI: 1.771-2.749). These associations remained significant after adjustment for potential confounders, although the effect sizes were attenuated. Notably, the effect of hs-CRP on increased CVD risk was more pronounced among individuals with higher AIP levels. Mediation analysis revealed that hs-CRP mediated 6.6% of the association between AIP and CVD (*P*=0.042), while AIP mediated 20.3% of the association in the reverse pathway (*P*=0.008). The RERI between AIP and hs-CRP for CVD was 0.141 (95% CI: -0.102 to 0.384), suggesting a possible positive additive interaction. The ROC analysis indicated that the combined model had better predictive performance for CVD than either marker alone (AUC = 0.590), with the best performance observed in stroke prediction (AUC = 0.615). Subgroup analyses confirmed consistent associations across demographic and clinical subgroups, except in individuals with prediabetes or diabetes.

**Conclusions:**

Elevated levels of AIP and hs-CRP were independently and jointly associated with an increased risk of cardiovascular disease, particularly stroke. The observed mutual mediation effects and potential additive interaction suggest that lipid metabolism and inflammation may be interconnected in the pathophysiological processes underlying cardiovascular risk. These findings highlight the potential value of incorporating both biomarkers into cardiovascular risk assessment models to enhance early identification and prevention strategies among middle-aged and older adults.

## Introduction

Cardiovascular diseases (CVD), including ischemic heart disease and stroke, are the leading cause of mortality and disease burden globally (1). The prevalence of CVD continues to rise globally, with total cases increasing from 271 million in 1990 to 523 million in 2019. During the same period, CVD-related deaths have grown by more than 6 million, reaching 18.6 million in 2019 (1). In China, CVD is the primary cause of death, accounting for 46.74% and 44.26% of mortality in rural and urban populations, respectively, in 2019 (2). Therefore, timely and accurate identification of high-risk CVD populations, alongside the implementation of effective preventive strategies, is critically important. Interestingly, despite China’s unique cultural traditions, dietary patterns, and healthcare landscape, recent studies have revealed a convergence in CVD risk factors between the Chinese and Western populations. This phenomenon may be attributed to rapid urbanization, a growing prevalence of sedentary lifestyles, and increasing consumption of high-fat, energy-dense diets. These changes mirror a broader global trend of declining physical activity levels and unhealthy metabolic profiles, highlighting the urgent need for updated risk prediction tools that are globally relevant yet population-specific.

Plasma lipid levels have been recognized as key risk factors and predictors of CVD (3) Dyslipidemia is primarily characterized by elevated levels of total cholesterol (TC), triglycerides (TG), low-density lipoprotein cholesterol (LDL-C), and reduced levels of high-density lipoprotein cholesterol (HDL-C) (4, 5). The atherogenic index of plasma (AIP) is a newly identified lipid metabolism-related biomarker of atherosclerosis, calculated as the log-transformed ratio of TG to HDL-C, measured in molar concentrations (6, 7). Growing evidence supports that AIP serves as a potential biomarker for atherosclerosis and CVD risk (8). Previous studies have suggested a positive association between high AIP and CVD risk, such as coronary heart disease and ischemic stroke, indicating that patients with high AIP are more susceptible to cardiovascular events (9–11). These studies have focused on the relationship between AIP levels and the incidence of cardiovascular diseases (e.g. myocardial infarction and coronary heart disease), as well as the prognosis of CVD patients. These findings suggest that AIP could serve as a valuable biomarker for assessing the risk or predicting the prognosis of CVD. Moreover, AIP is also closely associated with CVD risk factors such as insulin resistance (12), diabetes (13), hypertension (14), and metabolic syndrome (15).

Low-grade inflammation may be essential for the accelerated progression of atherosclerosis and CVD events (16). C-reactive protein (CRP) is an acute-phase reactant that plays a critical role in both acute and chronic inflammation (16). A single measurement of plasma CRP levels can predict future CVD events, such as myocardial infarction (MI) and stroke (17, 18). Substantial evidence supports high-sensitivity CRP (hs-CRP) as a clinical marker of inflammation to achieve risk stratification for CVD in clinical practice (19). Compared to risk scores based solely on traditional Framingham risk factors, prediction models incorporating hs-CRP demonstrate superior discrimination, calibration, and reclassification abilities in predicting new-onset CVD events (20).

Recent studies suggest that dysregulated lipid metabolism and inflammatory responses may promote each other through shared pathophysiological mechanisms (21, 22). Dyslipidemia may enhance the pro-inflammatory milieu by persistently activating circulating inflammatory cytokines and increasing the lipotoxicity of free fatty acids (21). Meanwhile, inflammation and cytokine-mediated alterations in lipase activity—such as changes in incretins, lipoprotein lipase, and cholesteryl ester transfer protein—may in turn drive pro-atherogenic lipid abnormalities (23). Oxidative stress may represent a key mechanistic link connecting lipid disorders, inflammation, and the progression of CVD events (24). Previous studies have demonstrated that the high triglyceride and low HDL-C levels reflected by AIP are associated with elevated oxidative stress (25, 26). This, in turn, contributes to endothelial dysfunction, lipid peroxidation, and activation of inflammatory pathways, thereby amplifying systemic inflammation.

Although there is biological plausibility supporting the interrelationship between AIP and hs-CRP, epidemiological evidence regarding their joint effect remains limited, and investigations into potential mediation pathways between them are even more scarce. To address this knowledge gap, we conducted a prospective study using a nationally representative cohort to assess the independent and combined associations of AIP and hs-CRP with incident CVD. Furthermore, mediation analysis was performed to explore their mutual influence on cardiovascular risk. This study aims to enhance the understanding of the interactive mechanisms between dyslipidemia and chronic inflammation in CVD pathogenesis, and to provide epidemiological evidence supporting improved risk stratification and the development of personalized, targeted prevention strategies.

## Methods

### Data sources and study population

**Figure 1:**
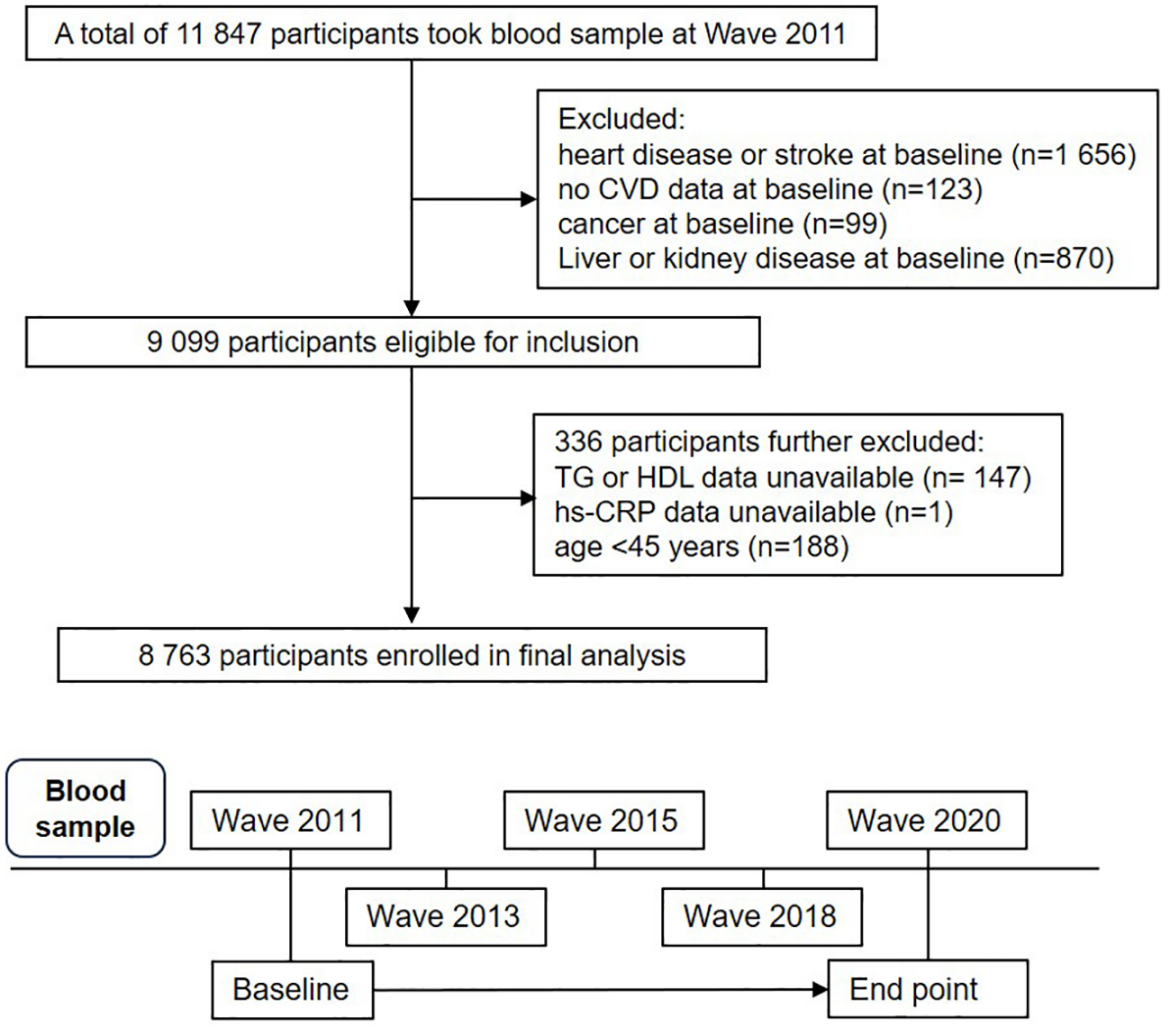
Flowchart and follow-up setting of this current study.

This study is a secondary analysis of the China Health and Retirement Longitudinal Study (CHARLS) (http://charls.pku.edu.cn/), a comprehensive national cohort study designed to represent the population across China. The study used a multi-stage stratified probability sampling method with sampling proportional to population size, recruiting participants from rural and urban areas across 150 counties or districts in 28 provinces in China. A total of 17,708 individuals from 10,257 households, aged 45 years or older, were recruited (27, 28). Data on sociodemographic characteristics, lifestyle behaviors, and health status were collected using standardized questionnaires (29). The CHARLS baseline survey began in 2011 (Wave 1). To date, CHARLS has released four waves of follow-up data: Wave 2 in 2013, Wave 3 in 2015, Wave 4 in 2018, and Wave 5 in 2020. In this study, 11,847 participants with blood samples from Wave 1 were included in the analysis. 2,255 participants were excluded for the following reasons: (1) self-reported CVD, cancer, liver disease and kidney disease at baseline; (2) lack of measurements for TG, HDL-C, or hs-CRP levels; (3) age under 45 years. A total of 8,763 eligible participants were ultimately included in the final analysis, with detailed inclusion and exclusion processes shown in **Figure 1**.

The CHARLS study was conducted by the principles of the Declaration of Helsinki and was approved by the Institutional Review Board of Peking University (IRB00001052-11015). Written informed consent was obtained from all participants. This study follows the Strengthening the Reporting of Observational Studies in Epidemiology (STROBE) guidelines for reporting observational studies.

### Exposure

Medical personnel from the Chinese Center for Disease Control and Prevention collected fasting venous blood samples according to standard protocols, which were subsequently tested in the central laboratory (30). Triglycerides and HDL-C were measured using enzymatic colorimetric methods. The within-assay coefficients of variation for triglycerides and HDL-C were 1.5% and 1.0%, respectively. Hs-CRP concentrations were measured using the immunoturbidimetric method. The AIP is defined as the logarithmic transformation of the molar concentration ratio of triglycerides to HDL-C, mathematically expressed as lg [TG (mmol/L)/HDL-C (mmol/L)] (7). Due to the lack of clinical cutoff points for AIP, the median value of AIP was used, where values above the median are interpreted as elevated. A hs-CRP level greater than 1 mg/L serves as the clinical threshold for indicating inflammation (31). Based on AIP (the median value of [-0.088] as the cutoff) and hs-CRP (1 mg/L as the cutoff), a joint analysis was performed to compare four groups of participants (AIP < median and hs-CRP < 1 mg/L, AIP < median and hs-CRP ≥ 1 mg/L, AIP ≥ median and hs-CRP < 1 mg/L and AIP ≥ median and hs-CRP ≥ 1 mg/L).

### Definition of outcomes

The primary outcome of this study was the incidence of CVD events during follow-up. Consistent with previous studies based on the CHARLS cohort, CVD was defined as a composite outcome, including both self-reported physician-diagnosed heart disease and stroke. Participants were asked the following standardized questions: (1) *“Have you been told by a doctor that you have been diagnosed with a heart attack, coronary heart disease, angina, congestive heart failure, or other heart problems?”*, and (2) *“Have you been told by a doctor that you have been diagnosed with a stroke?”* Participants who responded “yes” to either question were classified as having an incident CVD event.

### Covariates

At baseline, the study considered the following potential confounding variables. Demographic covariates included age, sex (male, female), education level (illiterate, primary, middle school, high school or above), residence (rural, urban), and marital status (currently married, others). Health behavior covariates included smoking status (yes/no), alcohol consumption (yes/no), and body mass index (BMI), calculated as weight (kg) divided by height squared (m²) (32). Household fuel use for cooking and heating was categorized as either clean fuels (natural gas, biogas, liquefied petroleum gas, electricity) or solid fuels (coal, crop residues, wood, charcoal). Physical activity (PA) was assessed using corresponding metabolic equivalent (MET) values and the scoring criteria of the IPAQ (33). Hypertension was defined as measured blood pressure ≥140/90 mmHg or self-reported diagnosis or treatment (34). Glycemic metabolic status (GMS) was classified according to ADA criteria into normal glucose regulation (NGR), prediabetes, and diabetes (35). Arthritis or rheumatism was defined based on self-reported physician diagnosis. Medication history, including use of antihypertensive, antidiabetic, and lipid-lowering drugs, was collected via standardized questionnaire. Frailty was defined by the Fried phenotype, with a score of 3 or higher indicating frailty (36, 37). For more detailed definitions, please refer to **Supplementary Table S1**. Some missing data existed in the CHARLS dataset, as detailed in **Table 1**. To minimize bias and enhance robustness, missing values were addressed using multiple imputation via the R package ‘mice’.

**Table 1:** Baseline characteristics of the study participants.

Characteristics	Total	AIP<median & hs-CRP<1mg/L	AIP ≥ median & hsCRP≥1mg/L.	*P value*
Participants, No.	8763	2484	2524	
Age, years (SD)	59.1 (9.6)	58.6 (9.6)	59.4 (9.4)	< 0.001
Sex, male, n (%)	4181 (47.7)	1170 (47.1)	1149 (45.5)	< 0.001
Residence, urban, n (%)	7187 (82.0)	2116 (85.2)	1938 (76.8)	< 0.001
Current married, n (%)	7738 (88.3)	2210 (89.0)	2225 (88.2)	0.001
Education, n (%)				< 0.001
Illiterate	2552 (29.1)	733 (29.5)	722 (28.6)	
Primary	3481 (39.7)	1016 (40.9)	946 (37.5)	
Middle school	1785 (20.4)	501 (20.2)	550 (21.8)	
High school+	945 (10.8)	234 (9.4)	306 (12.1)	
Smoking, n (%)	3426 (39.1)	945 (38.0)	964 (38.2)	< 0.001
Alcohol consumption, n (%)	2325 (26.5)	718 (28.9)	565 (22.4)	< 0.001
Fuel for cooking, n (%)				< 0.001
Clean fuels	1672 (19.1)	430 (17.3)	536 (21.2)	
Solid fuels	6361 (72.6)	1882 (75.8)	1740 (68.9)	
Missing	730 (8.3)	172 (6.9)	248 (9.8)	
Fuel for hearting, n (%)				< 0.001
Clean fuels	3772 (43.0)	1023 (41.2)	1197 (47.4)	
Solid fuels	4900 (55.9)	1435 (57.8)	1302 (51.6)	
Missing	91 (1.0)	26 (1.0)	25 (1.0)	
BMI, kg/m2				< 0.001
Continuous (SD)	23.1 (4.4)	22.0 (3.7)	24.7 (4.9)	
<23.9	5590 (63.8)	1916 (77.1)	1160 (46)	
24-27.9	2195 (25.0)	452 (18.2)	835 (33.1)	
≥28	882 (10.1)	95 (3.8)	491 (19.5)	
Hypertension, n (%)	3170 (36.2)	682 (27.5)	1202 (47.6)	< 0.001
NGR	5645 (65.1)	1864 (75.6)	1307 (52.5)	
Pre-diabetes	1620 (18.7)	384 (15.6)	564 (22.6)	
Diabetes	1403 (16.2)	216 (8.8)	620 (24.9)	
Dyslipidemia, n (%)	614 (7)	107 (4.3)	284 (11.3)	< 0.001
Arthritis or Rheumatism, n (%)	2748 (31.4)	735 (29.6)	828 (32.8)	0.066
Antidiabetic, n (%)	273 (3.1)	37 (1.5)	129 (5.1)	< 0.001
Lipid-lowering drugs, n (%)	310 (3.5)	49 (2.0)	164 (6.5)	< 0.001
Antihypertensive, n (%)	1427 (16.3)	233 (9.4)	640 (25.4)	< 0.001
Frailty, n (%)	386 (4.4)	91 (3.7)	127 (5.0)	0.108
Physical activity, n (%)				< 0.001
Low	1350 (37.6)	477 (46.3)	293 (28.4)	
Moderate	1167 (32.5)	280 (27.2)	400 (38.8)	
High	1073 (29.9)	273 (26.5)	337 (32.7)	
Total-C (SD), mg/dl	193.4 (38.6)	189.3 (35.1)	200.7 (41.7)	< 0.001
LDL-C (SD), mg/dl	116.3 (35.0)	115.0 (30.6)	119.3 (39.5)	< 0.001
HDL-C (SD), mg/dl	51.2 (15.3)	61.7 (14.1)	40.8 (9.7)	< 0.001
TG (IQR), mg/dl	105.3 (74.3-154.0)	75.2 (60.2-90.3)	158.4 (124.8-215.9)	< 0.001
HbA1c (IQR), %	5.1 (4.9-5.4)	5.1 (4.8-5.3)	5.2 (4.9-5.6)	< 0.001
hsCRP (IQR), mg/L	1.0 (0.5-2.1)	0.5 (0.4-0.7)	2.1 (1.4-3.8)	< 0.001

Data are presented as the mean ± SD, median (IQR), or number (%), as appropriate. SD, standard deviation; IQR, interquartile range; hs-CRP, high-sensitivity C-reactive protein; AIP, atherogenic Index of Plasma; BMI, body mass index; GMS, glycemic metabolic status; NGR, normal glucose regulation; HbA1c, glycated Hemoglobin; Total-C, total cholesterol; TG, triglyceride; LDL-C, low-density lipoprotein cholesterol; HDL-C, high-density lipoprotein cholesterol; N of missing: N of missing: smoking (n=4); alcohol consumption (n=6); education (n=10); BMI (n=1298); Fuel for cooking (n=730); Fuel for heating (n=91); Physical activity (n=5173).

### Statistical analyses

Continuous variables were presented as mean ± standard deviation or median (interquartile range), while categorical variables were expressed as counts (percentages). Group differences were assessed using t-tests, Wilcoxon tests, or chi-square tests as appropriate.

The follow-up time for each participant was calculated from the baseline survey date (2011–2012) to the date of CVD diagnosis or the end of follow-up (wave 5 in 2020), whichever occurred first. Participants were categorized based on combined levels of AIP (<median, ≥ median) and hs-CRP (<1mg/L, ≥1mg/). The Kaplan–Meier method was used to estimate the cumulative incidence of CVD, heart disease, and stroke across groups, and survival curves were compared to assess the effect of joint exposure.

Based on follow-up time, the incidence rate of new CVD events per 1,000 person-years was determined. To assess the association between AIP and hs-CRP and the risk of incident CVD, multivariable-adjusted Cox proportional hazards models were used to estimate hazard ratios (HRs) and 95% confidence intervals (CIs). The proportional hazards assumption was tested using the Schoenfeld residuals method, with no violations detected. Specifically, Model 1 was adjusted for age, sex, residence, marital status, and education level. Model 2 further adjusted for smoking status, alcohol consumption, frailty, and household fuel use based on Model 1. Model 3 additionally controlled for BMI level, hypertension, diabetes, arthritis or rheumatism, and medication history for hypertension, diabetes, and dyslipidemia based on Model 2.

The study conducted mediation analysis using the “mediation” package in R software. The mediator variable was modeled using linear regression, while the outcome variable was modeled using logistic regression. To control for confounding factors, three progressively adjusted multivariable models were constructed. The total effect was decomposed into the Average Direct Effect (ADE) and the Average Causal Mediation Effect (ACME), with the mediation proportion calculated as ACME/(ACME + ADE). All effects were estimated using the non-parametric bootstrap method with 1,000 resamples.

The relative excess risk due to interaction (RERI) was used to quantify the additive risk of joint exposure to both factors. RERI and its 95%CI were calculated from the corresponding models to determine the presence of additive interaction. Additionally, receiver operating characteristic (ROC) curve analysis was performed to assess the ability of combined AIP and hs-CRP to predict CVD risk. The discriminative performance of the combined model was evaluated by comparing the area under the curve (AUC) of models including single indicators versus the joint model. The ROC curves were constructed based on covariate-adjusted predictive models, and the optimal cutoff values were determined using the Youden index.

To evaluate the robustness and consistency of the results, the study conducted subgroup and multiple sensitivity analyses. Subgroup analyses stratified participants based on potential influencing factors including age, sex, education level, BMI, residence, hypertension status, and glucose metabolism status to assess the association between elevated AIP and hs-CRP levels and the CVD risk. Sensitivity analyses included: (1) defining low and moderate inflammatory states using hs-CRP cut-off values of 1.0 mg/L and 3.0 mg/L, respectively, and categorizing participants into six groups (31); (2) repeating the primary analysis in the complete dataset (n=6881); repeating the primary analysis after excluding participants with arthritis or rheumatism at baseline (n=6015) or history of medication use for hypertension, diabetes, and dyslipidemia (n=7082); (3) adjusting for physical activity in the subset with available physical activity data (n=2923); (4) comparing models adjusted for BMI versus waist circumference; (5) stratifying female participants by menopausal status and further adjusting for menopausal status to assess potential confounding; (6) repeating the primary analysis by replacing hs-CRP with alternative inflammatory biomarkers (white blood cell count, platelet count, and platelet-to-white blood cell ratio).

All statistical analyses were performed using R software (version 4.3.1). A two-tailed P-value < 0.05 was considered statistically significant.

## Results

### Baseline characteristics of study participants

**Figure 2:**
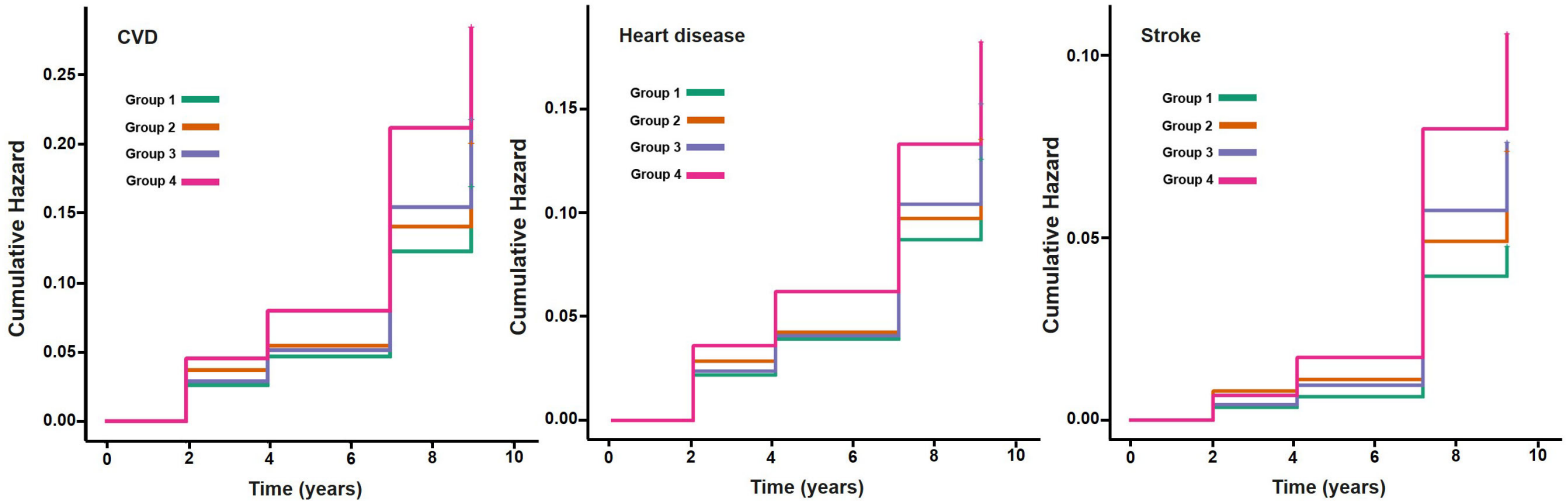
Kaplan-Meier plot of CVD by AIP and hs-CRP level. CVD, cardiovascular disease; AIP, atherogenic index of plasma, hs-CRP, high-sensitivity C-reactive protein; median of AIP: -0.088. Group1: AIP<median & hs-CRP<1mg/L; Group2: AIP <median & hs-CRP≥1mg/L; Group3: AIP ≥ median & hs-CRP<1mg/L; Group4: AIP ≥ median & hs-CRP≥1mg/L.

The final analysis included 8763 participants from the CHARLS study. The average age of participants was 59.1 (9.6) years, with 4181 males (47.7%). During a maximum follow-up of 9.0 years, 1693 participants (19.3%) developed incident CVD events, including 1217 cases of heart disease (13.9%) and 642 cases of stroke (7.3%). Compared with participants with AIP below the median and hs-CRP levels less than 1 mg/L, those with concurrently elevated AIP and hs-CRP levels were more likely to live in rural, had a higher level of education, and demonstrated a lower prevalence of alcohol consumption and solid fuel use, but significantly higher rates of hypertension, diabetes, dyslipidemia and obesity. In addition, they presented with significantly elevated levels of total cholesterol, LDL-C, triglycerides, and HbA1c, while HDL-C levels were lower. Baseline characteristics of all four participant groups are presented in **Supplementary Table S2**. The Kaplan-Meier cumulative incidence curves show that participants with both elevated AIP and hs-CRP levels (Group 4) had significantly higher risks of developing CVD, heart disease, and stroke throughout the follow-up period compared to other groups, suggesting that the combined elevation of AIP and hs-CRP may exacerbate the risk of cardiovascular events (**Figure 2**). **Supplementary Figure S1** further illustrates the K-M curves of cumulative CVD incidence stratified by three groups of hs-CRP (<1mg/L, 1-3mg/L, ≥ 3 mg/L).

### Association between AIP and hs-CRP with CVD

The impact of single exposures on CVD risk was first assessed, stratified by AIP index and hs-CRP levels (**Supplementary Table S3**). The risks of CVD events (including heart disease and stroke) were significantly elevated among participants with AIP ≥ median or hs-CRP ≥ 1 mg/L, with these associations remaining significant across adjusted models. **Table 2** shows the association of the co-exposure to AIP and hs-CRP with incident CVD events. The incidence of incident CVD increased from 17.7 cases per 1,000 person-years in Group 1 to 29.1 cases per 1,000 person-years in Group 4. In model 1, participants with both elevated AIP and hs-CRP levels (Group 4) had a 65.5% increased risk of CVD events compared to the reference group (HR=1.655, 95% CI: 1.455-1.883). This association remained statistically significant after full adjustment for all potential confounders (Model 3: HR=1.377, 95% CI: 1.192-1.589). Additionally, elevated hs-CRP alone (Group 3) was also significantly associated with increased CVD risk (HR=1.272, 95% CI: 1.092-1.482), whereas elevated AIP alone (Group 2) showed only a borderline association (Model 1: HR=1.159, 95% CI: 1.000-1.343). For stroke, the association was more pronounced, with a stepwise increase in stroke risk observed alongside elevated levels of AIP and hs-CRP. After comprehensive multivariable adjustment, participants in the Group 4 exhibited a 79.6% increased risk of stroke compared to the reference group (HR=1.796, 95% CI: 1.410-2.288). In contrast, the risk of heart disease was significantly elevated only among participants with concurrently high AIP and hs-CRP levels, but this association was attenuated or lost statistical significance after full adjustment. **Supplementary Table S4** illustrates the impact of co-exposure to the AIP index and hs-CRP levels on the incidence of CVD events when hs-CRP is divided into three groups (<1mg/L, 1-3mg/L, ≥ 3 mg/L). Regardless of hs-CRP levels, participants with higher AIP had a significantly increased risk of CVD events. Similarly, elevated hs-CRP levels were linked to higher CVD risk across AIP strata, with particularly strong associations observed for stroke (**Supplementary Table S5**).

**Table 2:** Risk of cardiovascular disease upon co-exposure stratified by AIP and hs-CRP.

	Model 1		Model 2		Model 3	
	HR (95% CI)	*P* value	HR (95% CI)	*P* value	HR (95% CI)	*P* value
Heart disease (cases/person-years)						
Group1 (295/21549)	1(Ref.)		1(Ref.)		1(Ref.)	
Group2 (239/16371)	1.064 (0.897, 1.263)	0.476	1.043 (0.870, 1.250)	0.651	1.010 (0.842, 1.211)	0.917
Group3 (263/16053)	1.203 (1.019, 1.421)	0.029	1.181 (0.990, 1.409)	0.065	1.106 (0.926, 1.322)	0.266
Group4 (420/21456)	1.402 (1.207, 1.628)	< 0.001	1.359 (1.158, 1.593)	<0.001	1.172 (0.991, 1.387)	0.064
Stroke (cases/person-years)						
Group1 (117/22096)	1(Ref.)		1(Ref.)		1(Ref.)	
Group2 (134/16791)	1.423 (1.110, 1.826)	0.005	1.486 (1.146, 1.927)	0.003	1.394 (1.074, 1.809)	0.012
Group3 (137/16456)	1.648 (1.287, 2.110)	< 0.001	1.796 (1.389, 2.323)	< 0.001	1.603 (1.237, 2.077)	<0.001
Group4 (254/22162)	2.207 (1.771, 2.749)	< 0.001	2.335 (1.853, 2.942)	< 0.001	2.045 (1.610, 2.602)	<0.001
CVD (cases/person-years)						
Group1 (377/21358)	1(Ref.)		1(Ref.)		1(Ref.)	
Group2 (336/16163)	1.159 (1.000, 1.343)	0.050	1.158 (0.992, 1.353)	0.064	1.114 (0.953, 1.302)	0.175
Group3 (369/15814)	1.355 (1.174, 1.565)	< 0.001	1.376 (1.182, 1.600)	< 0.001	1.272 (1.092, 1.482)	0.002
Group4(611/21001)	1.655 (1.455, 1.883)	< 0.001	1.651 (1.440, 1.893)	< 0.001	1.377 (1.192, 1.589)	< 0.001

HR, hazard ratio; CI, confidence interval; AIP, Atherogenic Index of Plasma; hs-CRP, high-sensitivity C-reactive protein; CVD, cardiovascular disease; Group1: AIP<median & hs-CRP<1mg/L; Group2: AIP <median & hs-CRP≥1mg/L; Group3: AIP ≥ median & hs-CRP<1mg/L; Group4: AIP ≥ median & hs-CRP≥1mg/L. Model 1: adjusted for age, sex, residence, marriage, education; Model 2: model 1 plus smoking, alcohol drinking, frailty and household fuel use; Model 3: model 2 plus BMI level, hypertension, diabetes, Arthritis or Rheumatism, and history of medication use for hypertension, diabetes, and dyslipidemia.

### Mediation analysis

**Figure 3:**
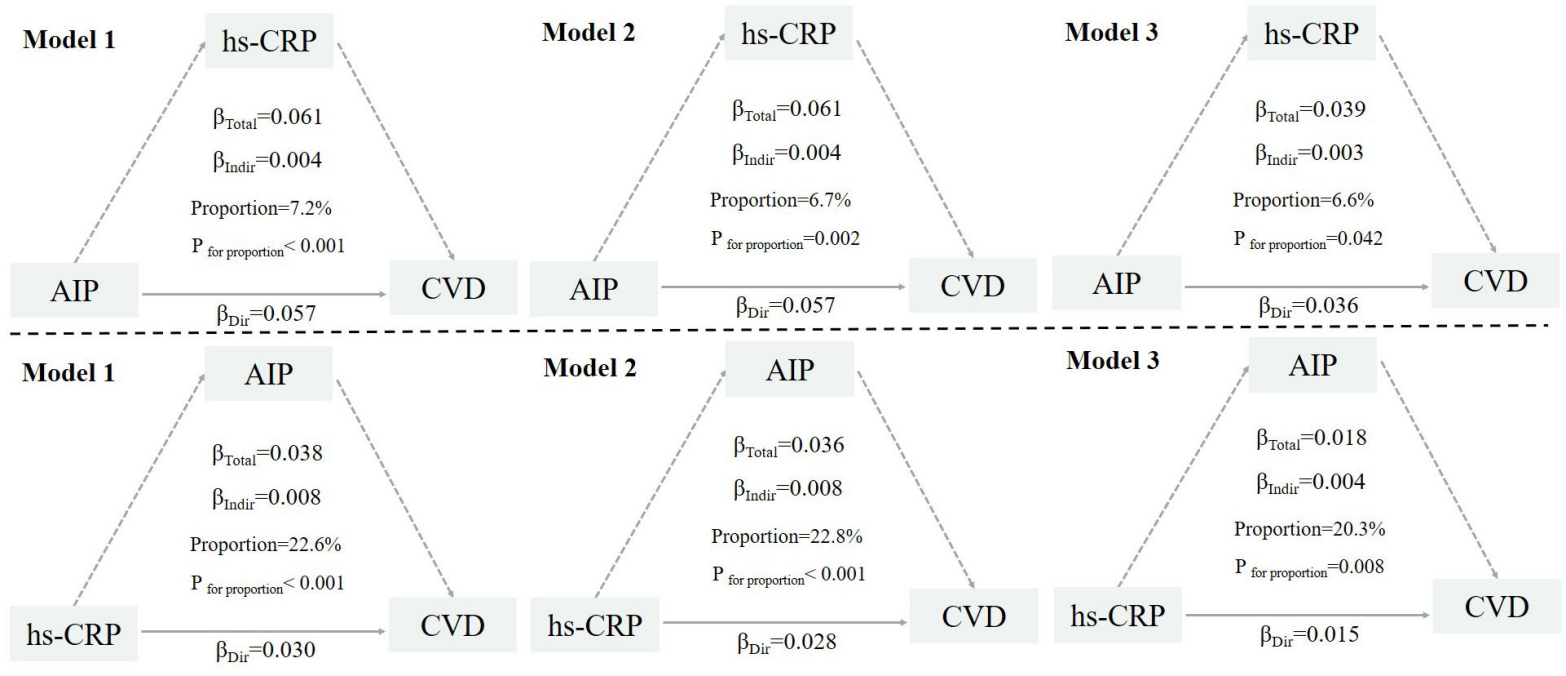
Mutual mediation effects of the AIP and hs-CRP on cardiovascular diseases. AIP, Atherogenic Index of Plasma; hs-CRP, high-sensitivity C-reactive protein; CVD, cardiovascular disease. Model 1: adjusted for age, sex, residence, marriage, education; Model 2: model 1 plus smoking, alcohol consumption, frailty and household fuel use; Model 3: model 2 plus BMI level, hypertension, diabetes, Arthritis or Rheumatism, and history of medication use for hypertension, diabetes, and dyslipidemia.

**Figure 3** illustrates the bidirectional mediation models between AIP and hs-CRP in relation to incident CVD events. In the fully adjusted model, hs-CRP significantly mediated the association between high AIP and the risk of CVD events, with a mediation proportion of 6.6% (*P* = 0.042). Similarly, AIP significantly mediated the relationship between high hs-CRP and CVD risk, with a mediation proportion of 20.3% (*P* = 0.008). For stroke, the mutual mediation effect remained significant, with proportions of 5.9% (*P* = 0.022) and 14.4% (*P* < 0.001), respectively. For heart disease, the mediation proportions were similar, at 7.5% (*P* = 0.041) and 29.8% (*P* = 0.040) (**Supplementary Figures S2**, **S3**).

### Relative excess risk due to interaction between AIP and hs-CRP

**Figure 4:**
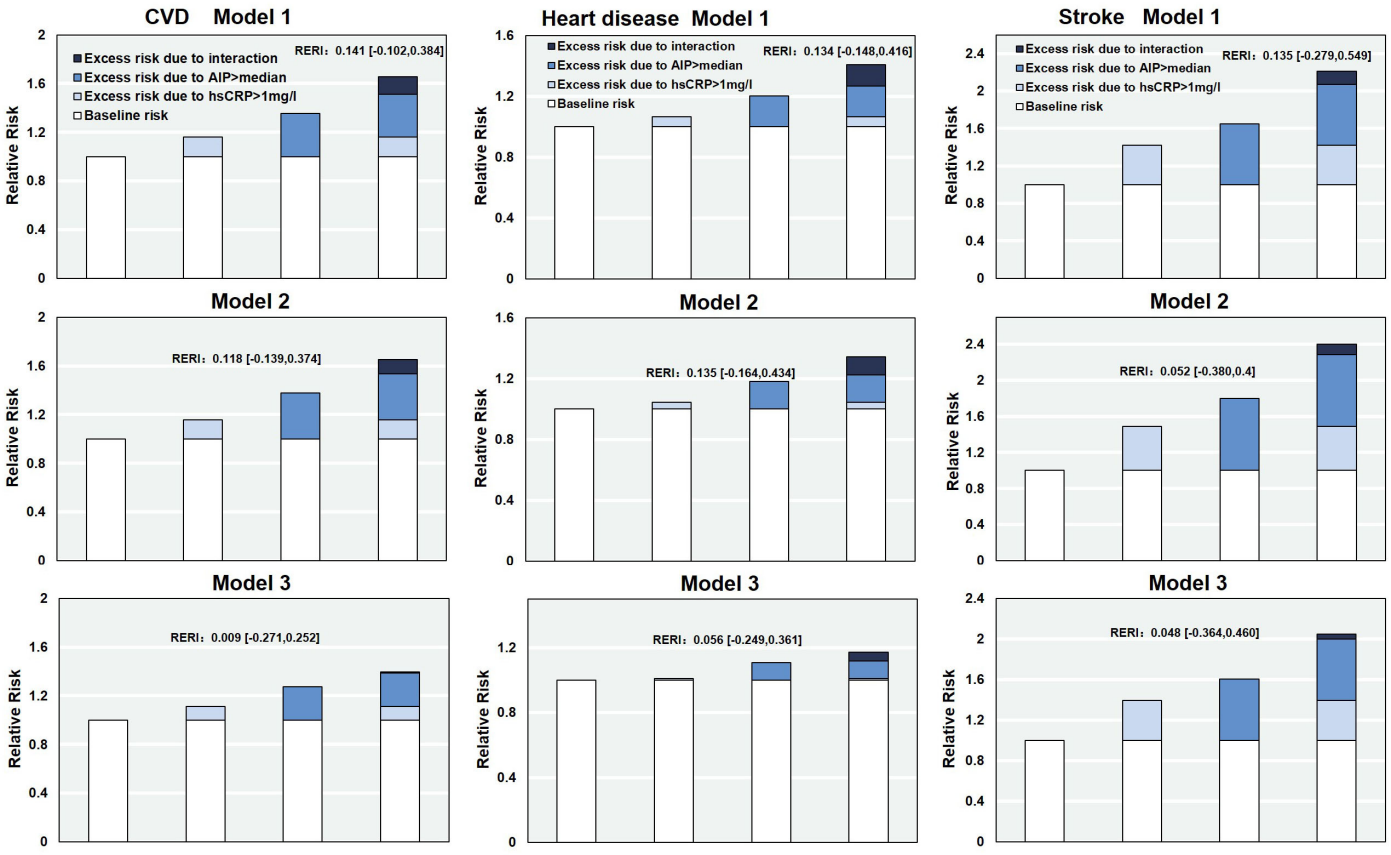
The relative excess risk due to the interaction of AIP and hs-CRP on CVD. RERI, Relative excess risk due to interaction; AIP, Atherogenic Index of Plasma; hs-CRP, high-sensitivity C-reactive protein; CVD, cardiovascular disease. Model 1: adjusted for age, sex, residence, marriage, education; Model 2: model 1 plus smoking, alcohol consumption, Frailty and household fuel use; Model 3: model 2 plus BMI level, hypertension, diabetes, Arthritis or Rheumatism, and history of medication use for hypertension, diabetes, and dyslipidemia.

A further analysis was conducted to evaluate the additive interaction between AIP and hs-CRP on the risk of CVD events. The RERI was 0.141 (95% CI: -0.102 to 0.384), suggesting a potential increase in CVD risk associated with the joint exposure to elevated AIP and hs-CRP levels. This finding indicates a trend toward supra-additive interaction. Similar additive interaction effects were observed for heart disease (RERI = 0.134; 95% CI: -0.148 to 0.416) and stroke (RERI = 0.135; 95% CI: -0.279 to 0.549), although the supra-addictive interaction was nonsignificant. After further adjustment for potential confounding factors, the additive interaction effect between AIP and hs-CRP was attenuated (**Figure 4**).

### ROC analysis of AIP and hs-CRP

**Figure 5:**
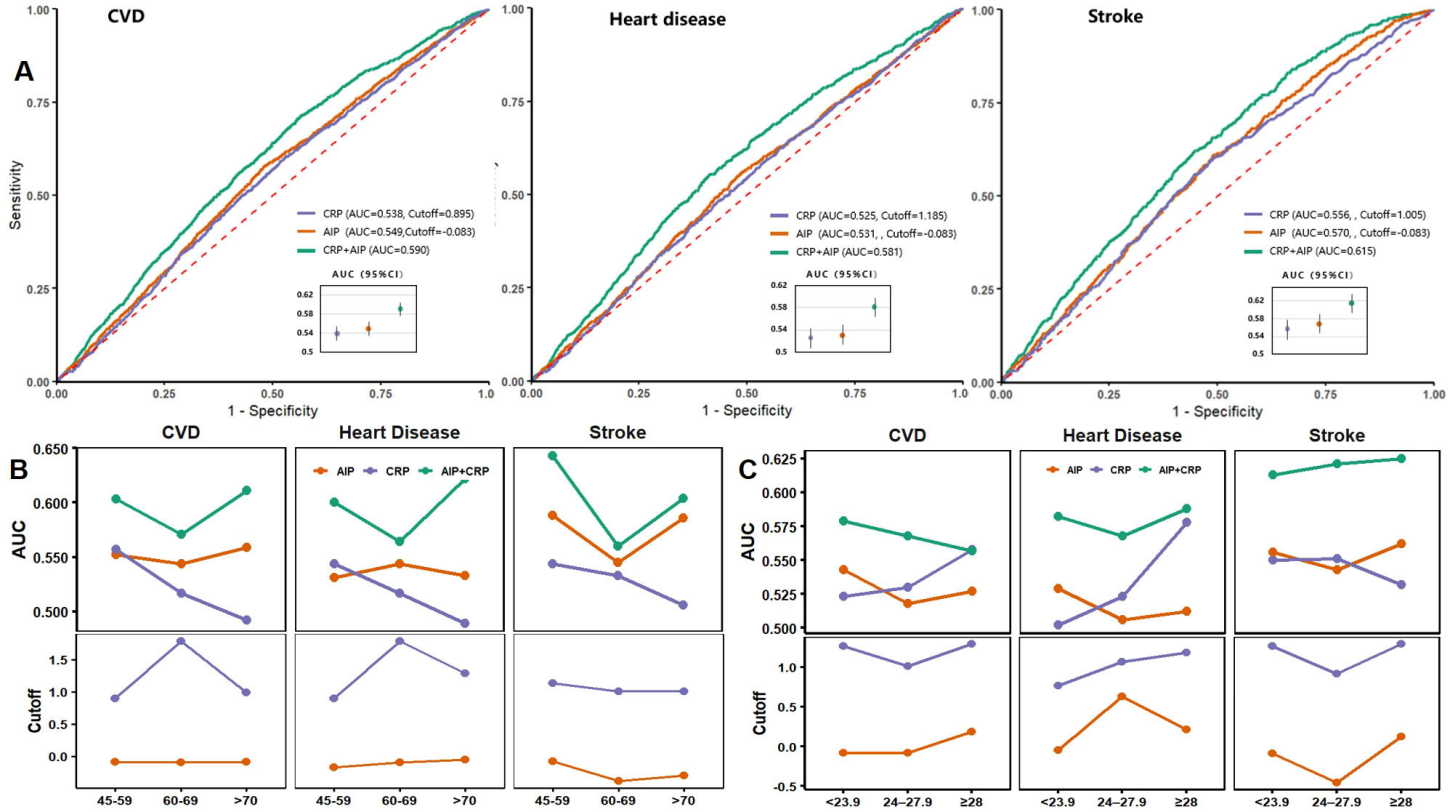
ROC curves and performance metrics for AIP, hsCRP, and their combination in predicting CVD. **(A)** ROC curves for CVD, heart disease, and stroke; **(B)** AUC and cutoff values stratified by age groups; **(C)** AUC and cutoff values stratified by BMI categories. AIP, Atherogenic Index of Plasma; hs-CRP, high-sensitivity C-reactive protein; CVD, cardiovascular disease; AUC, area under the curve.

ROC curves and model performance metrics for AIP, hs-CRP, and their combination in predicting CVD, heart disease, and stroke are presented in **Figure 5**. The combined AIP and hs-CRP model demonstrated superior discriminatory ability across all three outcomes, with AUCs of 0.590 (95% CI: 0.575–0.604) for CVD, 0.581 (95% CI: 0.564–0.598) for heart disease, and 0.615 (95% CI: 0.594–0.636) for stroke, all of which were higher than those of models using AIP or hs-CRP alone (**Figure 5A**). In the age-stratified analysis, the combined model consistently showed relatively high AUCs across age groups, with the best predictive performance for stroke observed in individuals aged 45–59 years (**Figure 5B**). BMI-stratified analysis revealed that the combined model (AIP and hs-CRP) achieved the highest AUC among participants with BMI ≥28 kg/m², suggesting stronger predictive capability in individuals with higher BMI (**Figure 5C**). Additionally, the optimal cutoff values for AIP and hs-CRP, determined using the Youden Index, varied slightly by age and BMI category but generally fluctuated around the predefined thresholds used in this study—namely, the median AIP value (–0.088) and 1 mg/L for hs-CRP. Detailed ROC curves stratified by age and BMI are provided in **Supplementary Tables S4**, **S5**.

### Subgroup analyses

Subgroup analyses stratified by potential risk factors were performed. As shown in **Table 3**, participants with elevated AIP and hs-CRP levels exhibited a higher risk of cardiovascular disease across subgroups, including age, sex, education level, BMI, residence, and hypertension status. Among individuals with normal glucose regulation, co-elevation of AIP and hs-CRP was significantly associated with increased CVD risk, whereas no significant association was observed in those with prediabetes or diabetes.

**Table 3:** Subgroup analyses of the association between AIP and hs-CRP and CVD.

	Group 1	Group 2	Group 3	Group 4	*P*-_Interaction_
Age (Case/Total)					0.282
45-59	1(Ref.) (195/1474)	1.108 (0.878, 1.398) (150/954)	1.173(0.947,1.454) (191/1175)	1.446 (1.179, 1.774) (310/1392)	
60-69	1(Ref.) (121/645)	1.201 (0.924, 1.561) (118/533)	1.351 (1.040, 1.754) (130/482)	1.251 (0.973, 1.608) (204/745)	
>70	1(Ref.) (61/365)	1.017 (0.711, 1.454) (68/409)	1.412 (0.951, 2.095) (48/202)	1.439 (1.014, 2.043) (97/387)	
Sex (Case/Total)					0.722
Male	1(Ref.) (167/1170)	1.080 (0.859, 1.357) (164/1017)	1.351 (1.072, 1.701) (159/845)	1.438 (1.157, 1.786) (257/1149)	
Female	1(Ref.) (210/1314)	1.147 (0.926, 1.421) (172/879)	1.203 (0.981, 1.476) (210/1014)	1.331 (1.097, 1.614) (354/1375)	
Education (Case/Total)					0.331
Illiterate	1(Ref.) (109/733)	1.296 (0.990, 1.697) (114/587)	1.518 (1.161, 1.985) (118/510)	1.465 (1.132, 1.896) (182/722)	
Primary	1(Ref.) (164/1016)	1.006 (0.791, 1.280) (130/779)	1.135 (0.896, 1.437) (145/740)	1.304 (1.043, 1.629) (230/946)	
Middle school	1(Ref.) (76/501)	0.978 (0.671, 1.425) (59/348)	1.139 (0.802, 1.617) (70/386)	1.507 (1.109, 2.048) (122/550)	
High school+	1(Ref.) (28/234)	1.915 (1.045, 3.509) (33/182)	1.547 (0.846, 2.831) (36/223)	2.220 (1.267, 3.891) (77/306)	
BMI (Case/Total)					0.240
<23.9	1(Ref.) (274/1916)	1.082 (0.899, 1.302) (218/1368)	1.387 (1.153, 1.669) (226/1146)	1.390 (1.156, 1.671) (246/1160)	
24-27.9	1(Ref.) (90/473)	1.027 (0.760, 1.389) (81/404)	0.960 (0.723, 1.275) (102/541)	1.294 (1.011, 1.657) (221/873)	
≥28	1(Ref.) (13/95)	1.944 (0.928, 4.071) (37/124)	1.916 (0.943, 3.893) (41/172)	2.053 (1.063, 3.965) (144/491)	
Residence (Case/Total)					0.618
Urban	1(Ref.) (307/2038)	1.083 (0.917, 1.279) (261/1531)	1.297 (1.103, 1.525) (290/1453)	1.390 (1.191, 1.622) (438/1836)	
Rural	1(Ref.) (36/262)	1.526 (0.974, 2.392) (41/204)	1.333 (0.852, 2.086) (42/229)	1.764 (1.198, 2.597) (97/423)	
Hypertension (Case/Total)					0.232
Yes	1(Ref.) (109/627)	1.342 (1.041, 1.732) (135/593)	1.502 (1.170, 1.928) (150/570)	1.669 (1.330, 2.096) (319/1071)	
No	1(Ref.) (234/1673)	1.013 (0.830, 1.237) (167/1142)	1.190 (0.979, 1.448) (182/1112)	1.251 (1.033, 1.516) (216/1188)	
GMS (Case/Total)					0.032
NGR	1(Ref.) (239/1726)	1.223 (1.015, 1.475) (210/1189)	1.347 (1.112, 1.630) (194/1058)	1.573 (1.313, 1.885) (270/1159)	
Pre-diabetes	1(Ref.) (65/359)	1.047 (0.731, 1.499) (57/303)	1.123 (0.796, 1.585) (68/324)	1.058 (0.764, 1.466) (117/511)	
Diabetes	1(Ref.) (39/198)	0.603 (0.373, 0.973) (31/219)	1.062(0.713, 1.581) (70/285)	0.993 (0.682, 1.446) (143/559)	

Model was adjusted for age, sex, residence, marriage, education, smoking, alcohol consumption, Frailty and household fuel use, BMI, hypertension, diabetes, Arthritis or Rheumatism, and history of medication use for hypertension, diabetes, and dyslipidemia. Group1: AIP<median & hs-CRP<1mg/L; Group2: AIP <median & hs-CRP≥1mg/L; Group3: AIP ≥ median & hs-CRP<1mg/L; Group4: AIP ≥ median & hs-CRP≥1mg/L.

### Sensitivity analyses

Multiple sensitivity analyses confirmed the robustness of the results. These included repeating the analysis in the complete dataset (n=6881), excluding participants with a history of medication use (n=7082), and excluding those with arthritis or rheumatism (n=6015). Additionally, adjustments for physical activity were made among 2923 participants with available data, all yielding consistent findings. Models adjusted for BMI and waist circumference showed similar results. Stratified analysis by menopausal status indicated a stronger association in premenopausal women, though no significant interaction was observed. Sensitivity analyses using white blood cell count, platelet count, and their ratio showed effects consistent with those observed with hs-CRP in combination with AIP on cardiovascular risk (see **Supplementary Tables S6**–**S10**).

## Discussion

After a maximum follow-up of 9 years among 8,763 Chinese adults aged 45 years and older, this study found that the combined effect of elevated AIP and hs-CRP levels significantly increased the risk of CVD, with a particularly pronounced impact on stroke. This association remained statistically significant even after comprehensive adjustment for demographic characteristics, lifestyle factors, comorbidities, and medication use, suggesting that the joint elevation of these biomarkers may constitute an independent and robust risk factor. Bidirectional mediation analysis further revealed significant causal mediation pathways between AIP and hs-CRP, each partially mediating their effects on CVD risk. Although the RERI did not reach statistical significance, the observed trend suggests a possible additive interaction contributing to additional risk. ROC analysis demonstrated that incorporating both AIP and hs-CRP into risk prediction models improved discriminative ability (AUC) compared to models using either marker alone. In summary, this study not only epidemiologically confirms the synergistic impact of AIP and hs-CRP on CVD risk but also provides mechanistic insights and predictive evidence supporting their combined use as intervention targets.

This study further corroborates the established association between elevated AIP levels and an increased risk of incident CVD events, particularly incident stroke. Previous research has extensively examined the positive correlation between individual AIP levels and cardiovascular risk. Research from the CHARLS and the Korean National Health Insurance Service-National Health Screening Cohort (NHIS-HEALS) has reported that elevated baseline AIP levels are significantly associated with an increased risk of stroke and CVD among diabetic patients (13, 38). Additionally, a large-scale prospective cohort study involving 54,123 participants, the Kailuan Study, reported that prolonged exposure to high AIP levels contributed to an increased risk of incident ischemic stroke, independent of traditional risk factors (39). Several studies have also highlighted the predictive value of AIP for fatty liver (40), obesity (41), diabetes (42), and glomerular filtration rate (43). The mechanism by which elevated AIP levels increase CVD risk likely involves its association with lipoprotein particle size, specifically its positive correlation with small, dense low-density lipoproteins (sd-LDL) (44). sd-LDL has several characteristics that make it more prone to atherosclerosis, including higher oxidation potential, enhanced binding to endothelial proteoglycans, increased permeability through the endothelial barrier, and greater uptake by macrophage scavenger receptors. These properties promote foam cell formation, which is a critical step in the early stages of atherosclerosis (45, 46).

Substantial epidemiological evidence has identified inflammation as a critical risk factor for CVD (47). Longitudinal studies indicate that elevated levels of pro-inflammatory markers in the blood, such as hs-CRP and IL-6, remain predictive of CVD risk among middle-aged and older adults, even after adjusting for other CVD risk factors (48, 49). These characteristics highlight the value of hs-CRP for CVD screening and effective risk stratification. The Emerging Risk Factors Collaboration (ERFC) analyzed data from 54 prospective studies, encompassing 160,309 participants, to assess the association between hs-CRP levels, CVD risk factors, and CVD risk. These results demonstrated that elevated CRP levels were significantly associated with increased risks of coronary artery disease, ischemic stroke, and cardiovascular mortality (50). After adjusting for Framingham risk variables, individuals with CRP levels above 3.0 mg/L had a 58% higher risk of coronary artery disease compared to those with CRP levels below 1.0 mg/L (51). For study population, on the one hand, elevated hs-CRP levels were associated with chronic low-grade systemic inflammation. On the other hand, hs-CRP may reflect underlying conditions such as liver disease, kidney disease, infections, and autoimmune disorders, which are also important contributors to increased hs-CRP. Efforts were made to minimize the influence of comorbidities by excluding major coexisting conditions where possible. However, at baseline, over 30% of participants self-reported having arthritis or rheumatic diseases. Although these individuals were not excluded from the analysis, relevant variables were adjusted for in the multivariable models to reduce potential confounding bias. Our findings showed that elevated hs-CRP levels were significantly associated with an increased CVD risk, particularly stroke. Some studies have indicated that postmenopausal status in women is associated with higher CRP levels (52). However, our study found that elevated hs-CRP levels were significantly associated with an increased risk of overall CVD, regardless of menopausal status. Mechanistically, chronic low-grade inflammation plays a central role in the development of atherosclerotic plaques, coronary artery disease, and ultimately myocardial infarction (53). Inflammatory stimuli promote the migration of monocytes into the vascular intima, where they differentiate into lipid-laden foam cells and contribute to plaque formation (54). Moreover, inflammation induces the expression of matrix metalloproteinases, inhibits collagen synthesis, and accelerates extracellular matrix degradation, leading to the thinning of the fibrous cap and formation of vulnerable plaques (54, 55). These unstable plaques are prone to rupture, triggering acute coronary syndromes. Additionally, a proinflammatory milieu enhances the expression of procoagulant factors, such as tissue factor, thereby promoting thrombogenesis and eventually leading to myocardial infarction (54).

Previous real-world prospective cohorts have demonstrated that co-exposure to elevated hs-CRP and high AIP is associated with an increased risk of diabetes (56). However, the association between joint exposure to AIP and hs-CRP with increased CVD risk, as well as their mutual mediation effects, has yet to be thoroughly explored. To our knowledge, this study is the first to examine the impact of joint exposure to atherogenic dyslipidemia and chronic inflammation on the risk of incident CVD and to investigate their potential mediating effects. This study has shown that simultaneous elevation of hs-CRP and AIP is significantly associated with an increased overall CVD, particularly stroke. Moreover, this association is more pronounced among participants with higher baseline AIP levels, suggesting that the impact of systemic inflammation on CVD risk may be partially dependent on dyslipidemia. This association may involve the following biological mechanisms: hs-CRP can directly bind to oxidized lipids associated with atherosclerosis and accumulate in lipid-rich arterial plaques. In addition, hs-CRP may enhance monocyte adhesion and migration to the vascular wall, as well as promote macrophage polarization, thereby facilitating the accumulation and expansion of macrophages in adipose tissue and atherosclerotic lesions (57, 58). In addition, oxidative stress is considered a key mechanism linking systemic inflammation, lipid abnormalities, and the development of cardiovascular disease (59, 60).The pathogenesis of inflammation is closely related to oxidative stress, with Nrf2, NLRP3, and JNK/ERK playing important roles in this process (61). As critical effector molecules initiating both inflammation and oxidative stress, JNK/ERK have become targets for clinical drug development (61). Moreover, oxidative stress can activate the NF-κB pathway, leading to the release of inflammatory mediators and resulting in lipid abnormalities and insulin resistance (62). Additionally, oxidative stress affects lipid metabolism by promoting fatty acid synthesis, inhibiting lipolysis, and disrupting lipid transport and lipoprotein metabolism, causing lipid peroxidation, metabolic disorders, and ectopic lipid accumulation. At the same time, dysregulation of cytokines and adipokines secreted by adipose tissue impacts insulin signaling pathways, thereby creating a vicious cycle of inflammation, oxidative stress and cardiac metabolic disease (61, 63, 64).

This study found that among individuals with normal glucose regulation, the combined elevation of the lipid index AIP and the inflammatory marker hs-CRP was significantly associated with an increased risk of CVD; however, this combined effect was not significant in individuals with prediabetes or diabetes. Several factors may explain this phenomenon. First, individuals with prediabetes and diabetes already have a high baseline risk of CVD, which may lead to a risk saturation effect, making it difficult to detect the marginal risk contribution of AIP and hs-CRP. Results from the AusDiab study indicated that individuals with prediabetes had a significantly higher risk of CVD incidence, with a relative risk of 2.5 (95% CI: 1.2–5.1) compared to those with normal glucose regulation (65). Second, patients with diabetes often receive various treatments, including glucose-lowering, lipid-lowering, and anti-inflammatory medications, which may attenuate the association between AIP, hs-CRP, and CVD risk. Additionally, the smaller sample size and fewer events in the prediabetes and diabetes groups may result in insufficient statistical power to detect a significant combined effect. These findings suggest that the combined AIP and hs-CRP indicator has greater predictive value for CVD risk assessment in individuals with normal glucose metabolism, whereas in those with abnormal glucose metabolism, a comprehensive evaluation incorporating more clinical factors is warranted.

## Strengths and limitations

The strengths of this study include the use of a large-scale, nationally representative cohort study and an analysis adjusted for multiple confounding variables and subgroup analyses to demonstrate the association between joint exposure to AIP and hs-CRP and future CVD risk. These findings suggest that AIP and hs-CRP can serve as cost-effective biomarkers for identifying future CVD risk in clinical decision-making. However, several limitations of this study should be acknowledged. First, as the data were derived from an observational study, we cannot confirm a causal relationship between atherogenic dyslipidemia, inflammatory markers, and cardiovascular risk. Nevertheless, AIP and hs-CRP are widely validated predictors of CVD events, and the primary aim of this study was to evaluate the co-exposure effect and mutual mediation between AIP and hs-CRP on cardiovascular risk. Second, although we adjusted for major potential CVD risk factors, residual or unmeasured confounding biases may still be present, such as differences in dietary patterns and environmental factors across regions. Third, due to the absence of medical records in the CHARLS dataset, information on chronic disease histories—including heart disease, stroke, hypertension, and diabetes—relied primarily on participants’ self-reports, which may introduce recall bias and disease misclassification, thereby affecting the accuracy of risk assessment. Fourth, the lack of information on the manufacturers and countries of origin of the measurement instruments and assay kits used for hs-CRP and other biomarkers in the CHARLS study may limit the reproducibility of our findings. Finally, as this study was limited to a Chinese population, these findings may not be generalizable to other racial groups. Despite these limitations, these results provide clinical relevance, offering further insights into CVD risk stratification and early intervention.

## Conclusion

This study is the first to demonstrate a significant association between the co-exposure to atherogenic dyslipidemia and chronic inflammation and the incidence of CVD in a prospective, national cohort of Chinese adults. Furthermore, there is a substantial mutual mediation and potential additive interaction effect between atherogenic dyslipidemia and chronic inflammation on cardiovascular risk. These findings underscore the importance of integrated assessment of AIP and inflammatory markers to identify individuals at risk of CVD and to develop early, proactive prevention strategies. Additionally, it is reasonable to hypothesize that dual-targeted interventions addressing both dyslipidemia and inflammation could provide clinical benefits that exceed the effects of targeting each factor individually.

## Data Availability

The original contributions presented in the study are included in the article/**Supplementary Material**. Further inquiries can be directed to the corresponding author.
