# Supplementary material for: Association of the atherogenic index of plasma and high-sensitivity C-reactive protein with incident cardiovascular disease: evidence from a national cohort of middle-aged and older Chinese adults
Source: Front Endocrinol (Lausanne). 2025 Aug 6;16:1618157. doi: 10.3389/fendo.2025.1618157 (PMC12364642; doi:10.3389/fendo.2025.1618157)

**Supplementary File**

**Association of The Atherogenic Index of Plasma and high-sensitivity C-reactive protein with Incident Cardiovascular Disease: Evidence from a National Cohort of Middle-Aged and Older Chinese Adults**

Table S1. Specific definitions of health conditions and functional status.

Table S2. Baseline characteristics of all participants.

Table S3: Risk of CVD upon individual exposure stratified by AIP index and hs-CRP.

Table S4: Risk of CVD upon co-exposure stratified by AIP and hs-CRP.

Table S5. Risk reclassification of cardiovascular disease based on AIP and hs-CRP.

Table S6: Sensitivity analyses of co-exposure for AIP and hs-CRP on CVD events.

Table S7: Comparison of models adjusted for waist circumference versus BMI in relation to CVD events.

Table S8: Stratified analyses of AIP and hs-CRP in relation to cardiovascular risk according to menopausal status in female participants.

Table S9. Multivariable models additionally adjusted for menopausal status in female participants.

Table S10: Sensitivity analyses of co-exposure for AIP and other inflammatory biomarkers on CVD events.

Figure S1: Cumulative Hazard of CVD by AIP and hs-CRP level.

Figure S2. Mutual mediation effects of AIP and hs-CRP on heart disease.

Figure S3. Mutual mediation effects of AIP and hs-CRP on stroke.

Figure S4. ROC curves for AIP, hs-CRP, and their combination in predicting CVD stratified by age groups.

Figure S5. ROC curves for AIP, hs-CRP, and their combination in predicting CVD stratified by BMI levels.

Figure S6. Distribution Characteristics of AIP and hs-CRP.

Table S1. **Specific definitions of health conditions and functional status.**

| Diabetes (ADA) | Fasting blood glucose ≥ 126 mg/dL (≥7.0mmol/l) or HbA1c ≥ 6.5%; self-reported diagnosis of diabetes; use of diabetes medication. |
| --- | --- |
| Prediabetes (ADA) | Fasting blood glucose: ≥110, <126 mg/dL (≥6.1, <7.0mmol/l) or HbA1c: 5.7%-6.4% and without self-reported diagnosis of diabetes, use of diabetes medication. |
| NGR (ADA) | Fasting blood glucose < 110 mg/dL (<6.1 mmol/L), HbA1c < 5.7%, without a diagnosis of diabetes or Prediabetes; without use of diabetes medication. |
| Hypertension | SBP ≥140 mmHg or DBP ≥90 mmHg; self-reported diagnosis of hypertension; use of antihypertensive medications. |
| Arthritis or rheumatism | self-reported diagnosis of Arthritis or rheumatism |
| Frailty | Meet any three or more of the five:  ① weight loss: self-reported ≥5 kg weight loss in past year or BMI≤18.5 kg/m²;  ②exhaustion: feeling everything was an effort or unable to get going last week (per CES-D);  ③ low physical activity: no physical activity or walking ≥10 min in a usual week;  ④ slow walking speed: difficulty walking 100 m or climbing several flights of stairs without resting;  ⑤ low grip strength: difficulty lifting or carrying objects heavier than 5 kg. |
| Dyslipidemia | Self-reported physician-diagnosed, and/or current use of lipid-lowering drugs, and/or TC≥ 240 mg/dl, TG ≥ 150 mg/dl, HDL-C < 40 mg/dl, LDL-C ≥ 160 mg/dl; |
| Physical activity (PA) | 1. PA Level Classification: Low: <600MET-minutes/week; Moderate:600-3000 MET-minutes/week; High: > 3000 MET-minutes/week; 2. PA Type Classification: Vigorous PA: MET = 8.0, e.g., climbing, running, farming; Moderate PA: MET = 4.0, e.g., brisk walking, Tai Chi; Light PA: MET = 3.3, e.g., casual walking; 3. Daily PA Duration Categories: 0 min; 10-29 min; 30-119 min; 120-239 min; ≥240 min; 4. PA Calculation Formula: MET-minutes/week=MET×days per week×duration (minutes/day midpoint). |

Table S2. Baseline characteristics of all participants.

| Characteristics | Total | Group 1 | Group 2 | Group 3 | Group 4 | *P value* |
| --- | --- | --- | --- | --- | --- | --- |
| Participants, No. | 8 763 | 2484 | 1896 | 1859 | 2524 |  |
| Age, years (SD) | 59.1 (9.6) | 58.6 (9.6) | 60.8 (10.2) | 57.5 (8.9) | 59.4 (9.4) | < 0.001 |
| Sex, male, n (%) | 4181 (47.7) | 1170 (47.1) | 1017 (53.6) | 845 (45.5) | 1149 (45.5) | < 0.001 |
| Residence, urban, n (%) | 7187 (82.0) | 2116 (85.2) | 1606 (84.7) | 1527 (82.1) | 1938 (76.8) | < 0.001 |
| Current married, n (%) | 7738 (88.3) | 2210 (89.0) | 1630 (86.0) | 1673 (90.0) | 2225 (88.2) | 0.001 |
| Education, n (%) |  |  |  |  |  | < 0.001 |
| Illiterate | 2552 (29.1) | 733 (29.5) | 587 (31) | 510 (27.4) | 722 (28.6) |  |
| Primary | 3481 (39.7) | 1016 (40.9) | 779 (41.1) | 740 (39.8) | 946 (37.5) |  |
| Middle school | 1785 (20.4) | 501 (20.2) | 348 (18.4) | 386 (20.8) | 550 (21.8) |  |
| High school+ | 945 (10.8) | 234 (9.4) | 182 (9.6) | 223 (12) | 306 (12.1) |  |
| Smoking, n (%) | 3426 (39.1) | 945 (38.0) | 839 (44.3) | 678 (36.5) | 964 (38.2) | < 0.001 |
| Alcohol consumption, n (%) | 2325 (26.5) | 718 (28.9) | 588 (31) | 454 (24.4) | 565 (22.4) | < 0.001 |
| Fuel for cooking, n (%) |  |  |  |  |  | < 0.001 |
| Clean fuels | 1672 (19.1) | 430 (17.3) | 342 (18) | 364 (19.6) | 536 (21.2) |  |
| Solid fuels | 6361 (72.6) | 1882 (75.8) | 1413 (74.5) | 1326 (71.3) | 1740 (68.9) |  |
| Missing | 730 (8.3) | 172 (6.9) | 141 (7.4) | 169 (9.1) | 248 (9.8) |  |
| Fuel for hearting, n (%) |  |  |  |  |  | < 0.001 |
| Clean fuels | 3772 (43.0) | 1023 (41.2) | 790 (41.7) | 762 (41) | 1197 (47.4) |  |
| Solid fuels | 4900 (55.9) | 1435 (57.8) | 1081 (57) | 1082 (58.2) | 1302 (51.6) |  |
| Missing | 91 (1.0) | 26 (1.0) | 25 (1.3) | 15 (0.8) | 25 (1.0) |  |
| BMI, kg/m2 |  |  |  |  |  | < 0.001 |
| Continuous (SD) | 23.1 (4.4) | 22.0 (3.7) | 22.4 (4.1) | 23.3 (4.2) | 24.7 (4.9) |  |
| <23.9 | 5590 (63.8) | 1916 (77.1) | 1368 (72.2) | 1146 (61.6) | 1160 (46) |  |
| 24-27.9 | 2195 (25.0) | 452 (18.2) | 387 (20.4) | 521 (28.0) | 835 (33.1) |  |
| ≥28 | 882 (10.1) | 95 (3.8) | 124 (6.5) | 172 (9.3) | 491 (19.5) |  |
| Hypertension, n (%) | 3170 (36.2) | 682 (27.5) | 656 (34.6) | 630 (33.9) | 1202 (47.6) | < 0.001 |
| Diabetes, n (%) | 623 (7.1) | 82 (3.3) | 114 (6.0) | 125 (6.7) | 302 (12.0) | < 0.001 |
| GMS |  |  |  |  |  | < 0.001 |
| NGR | 5645(65.1) | 1864(75.6) | 1300(69.5) | 1174(63.7) | 1307(52.5) |  |
| Pre-DM | 1620(18.7) | 384(15.6) | 319(17) | 353(19.2) | 564(22.6) |  |
| DM | 1403(16.2) | 216(8.8) | 252(13.5) | 315(17.1) | 620(24.9) |  |
| Dyslipidemia | 614 (7) | 107 (4.3) | 89 (4.7) | 134 (7.2) | 284 (11.3) | < 0.001 |
| Arthritis or Rheumatism, n (%) | 2748 (31.4) | 735 (29.6) | 613 (32.3) | 572 (30.8) | 828 (32.8) | 0.066 |
| Antidiabetic, n (%) | 273 (3.1) | 37 (1.5) | 51 (2.7) | 56 (3.0) | 129 (5.1) | < 0.001 |
| Lipid-lowering drugs | 310 (3.5) | 49 (2.0) | 37 (2.0) | 60 (3.2) | 164 (6.5) | < 0.001 |
| Antihypertensive, n (%) | 1427 (16.3) | 233 (9.4) | 266 (14) | 288 (15.5) | 640 (25.4) | < 0.001 |
| Frailty, n (%) | 386 (4.4) | 91 (3.7) | 89 (4.7) | 79 (4.2) | 127 (5.0) | 0.108 |
| Physical activity, n (%) |  |  |  |  |  | < 0.001 |
| Low | 1350 (37.6) | 477 (46.3) | 314 (40.3) | 266 (35.5) | 293 (28.4) |  |
| Moderate | 1167 (32.5) | 280 (27.2) | 233 (29.9) | 254 (33.9) | 400 (38.8) |  |
| High | 1073 (29.9) | 273 (26.5) | 233 (29.9) | 230 (30.7) | 337 (32.7) |  |
| Total-C (SD), mg/dl | 193.4 (38.6) | 189.3 (35.1) | 188.5 (36.9) | 193.7(39.0) | 200.7(41.7) | < 0.001 |
| LDL-C (SD), mg/dl | 116.3 (35.0) | 115.0 (30.6) | 115.9 (33.1) | 114.4 (35.6) | 119.3 (39.5) | < 0.001 |
| HDL-C (SD), mg/dl | 51.2 (15.3) | 61.7 (14.1) | 59.1 (13.8) | 43.5 (10.2) | 40.8 (9.7) | < 0.001 |
| TG (IQR), mg/dl | 105.3(74.3-154.0) | 75.2 (60.2-90.3) | 74.3 (61.1-89.6) | 148.7(122.1-197.4) | 158.4(124.8-215.9) | < 0.001 |
| HbA1c (IQR), % | 5.1 (4.9-5.4) | 5.1 (4.8-5.3) | 5.1 (4.9-5.4) | 5.1 (4.8-5.4) | 5.2 (4.9-5.6) | < 0.001 |
| hsCRP (IQR), mg/L | 1.0 (0.5-2.1) | 0.5 (0.4-0.7) | 2.1 (1.4-4.1) | 0.6 (0.4-0.8) | 2.1 (1.4-3.8) | < 0.001 |

Data are presented as the mean±SD, median (IQR), or number (%), as appropriate. Abbreviations: SD, standard deviation; IQR, interquartile range; hs-CRP: high-sensitivity C-reactive protein; AIP: Atherogenic Index of Plasma; BMI: body mass index; ADL, activities of daily living; IADL, instrumental activities of daily living; HbA1c, glycated Hemoglobin, Total-C, total cholesterol, TG, triglyceride, HDL-C, high-density lipoprotein cholesterol; LDL-C, low-density lipoprotein cholesterol. N of missing: smoking(n=4); alcohol consumption (n=6); education(n=10); BMI (n=1298); Fuel for cooking (n=730); Fuel for heating (n=91); Physical activity (n=5173). Group1: AIP<median & hs-CRP<1mg/L; Group2: AIP <median & hs-CRP≥1mg/L; Group3: AIP ≥ median & hs-CRP<1mg/L; Group4: AIP ≥ median & hsCRP≥1mg/L.

**Table S3:** Risk of CVD upon individual exposure stratified by AIP index and hs-CRP.

|  | Model 1 | | Model 2 | | Model 3 | |
| --- | --- | --- | --- | --- | --- | --- |
|  | HR (95% CI) | P value | HR (95% CI) | P value | HR (95% CI) | P value |
| AIP < median | 1 (Ref.) |  | 1 (Ref.) |  | 1 (Ref.) |  |
| AIP ≥ median |  |  |  |  |  |  |
| Heart disease | 1.283 (1.145, 1.437) | < 0.001 | 1.260 (1.116, 1.423) | <0.001 | 1.138 (1.003, 1.292) | 0.045 |
| Stroke | 1.662 (1.416, 1.949) | < 0.001 | 1.737 (1.470, 2.052) | < 0.001 | 1.448 (1.217, 1.722) | < 0.001 |
| CVD | 1.430 (1.297, 1.576) | < 0.001 | 1.436 (1.296, 1.592) | < 0.001 | 1.266 (1.137, 1.408) | < 0.001 |
| hs-CRP < 1mg/L | 1 (Ref.) |  | 1 (Ref.) |  | 1 (Ref.) |  |
| hs-CRP ≥ 1mg/L |  |  |  |  |  |  |
| Heart disease | 1.158 (1.034, 1.297) | 0.011 | 1.135 (1.006, 1.282 | 0.040 | 1.050 (0.927, 1.188) | 0.442 |
| Stroke | 1.464 (1.249, 1.717) | < 0.001 | 1.472 (1.248, 1.737) | <0.001 | 1.271 (1.073, 1.505) | 0.005 |
| CVD | 1.252 (1.137, 1.379) | < 0.001 | 1.240 (1.120, 1.374) | <0.001 | 1.123 (1.011, 1.246) | 0.030 |

Abbreviations: HR, hazard ratio; CI, confidence interval; BMI, body mass index; hs-CRP: high-sensitivity C-reactive protein; CVD: cardiovascular disease; AIP: Atherogenic Index of Plasma. Model 1: adjusted for age, sex, residence, marriage, education; Model 2: model 1 plus smoking, alcohol consumption, frailty and household fuel use; Model 3: model 2 plus BMI level, hypertension, diabetes, Arthritis or Rheumatism, and history of medication use for hypertension, diabetes, and dyslipidemia.

**Table S4:** Risk of CVD upon co-exposure stratified by AIP and hs-CRP

|  | Model 1 | | Model 2 | | Model 3 | |
| --- | --- | --- | --- | --- | --- | --- |
|  | HR (95% CI) | P value | HR (95% CI) | P value | HR (95% CI) | P value |
| Heart disease |  |  |  |  |  |  |
| Group1 | Ref |  |  |  |  |  |
| Group2 | 1.100 (0.908, 1.334) | 0.330 | 1.098 (0.896, 1.346) | 0.367 | 1.064 (0.868, 1.304) | 0.553 |
| Group3 | 0.996 (0.775, 1.279) | 0.972 | 0.939 (0.719, 1.227) | 0.644 | 0.908 (0.694, 1.186) | 0.478 |
| Group4 | 1.204 (1.019, 1.422) | 0.029 | 1.182 (0.991, 1.410) | 0.064 | 1.106 (0.926, 1.322) | 0.267 |
| Group5 | 1.425 (1.210, 1.679) | <0.001 | 1.438 (1.209, 1.710) | <0.001 | 1.256 (1.048, 1.504) | 0.014 |
| Group6 | 1.356 (1.106, 1.662) | 0.003 | 1.205 (0.963, 1.508) | 0.103 | 1.011 (0.802, 1.274) | 0.929 |
| Stroke |  |  |  |  |  |  |
| Group1 | Ref |  |  |  |  |  |
| Group2 | 1.528 (1.162, 2.008) | 0.002 | 1.598 (1.201, 2.127) | 0.001 | 1.514 (1.137, 2.016) | 0.005 |
| Group3 | 1.236 (0.867, 1.761) | 0.241 | 1.292 (0.896, 1.862) | 0.170 | 1.190 (0.825, 1.717) | 0.352 |
| Group4 | 1.649 (1.288, 2.111) | <0.001 | 1.797 (1.390, 2.323) | <0.001 | 1.604 (1.238, 2.078) | <0.001 |
| Group5 | 2.215 (1.747, 2.808) | <0.001 | 2.305 (1.795, 2.960) | <0.001 | 1.799 (1.387, 2.333) | <0.001 |
| Group6 | 2.191 (1.658, 2.895) | <0.001 | 2.391 (1.791, 3.193) | <0.001 | 1.790 (1.327, 2.414) | <0.001 |
| CVD |  |  |  |  |  |  |
| Group1 | Ref |  |  |  |  |  |
| Group2 | 1.213 (1.029, 1.431) | 0.021 | 1.237 (1.041, 1.471) | 0.016 | 1.191 (1.001, 1.416) | 0.049 |
| Group3 | 1.057 (0.853, 1.310) | 0.614 | 1.015 (0.808, 1.274) | 0.900 | 0.973 (0.775, 1.222) | 0.816 |
| Group4 | 1.356 (1.174, 1.565) | <0.001 | 1.376 (1.183, 1.601) | <0.001 | 1.272 (1.092, 1.482) | 0.002 |
| Group5 | 1.685 (1.465, 1.939) | <0.001 | 1.726 (1.488, 2.001) | <0.001 | 1.458 (1.250, 1.701) | <0.001 |
| Group6 | 1.596 (1.344, 1.896) | <0.001 | 1.509 (1.253, 1.816) | <0.001 | 1.222 (1.009, 1.480) | 0.040 |

Abbreviations: HR, hazard ratio; CI, confidence interval; BMI, body mass index; AIP: Atherogenic Index of Plasma; hs-CRP: high-sensitivity C-reactive protein; CVD: cardiovascular disease;

Model 1: adjusted for age, sex, residence, marriage, education;

Model 2: model 1 plus smoking, alcohol consumption, frailty and household fuel use;

Model 3: model 2 plus BMI level, hypertension, diabetes, Arthritis or Rheumatism, and history of medication use for hypertension, diabetes, and dyslipidemia.

Group1: AIP<median & hs-CRP<1mg/L; Group2: AIP <median & hs-CRP 1-3 mg/L; Group3: AIP <median & hs-CRP≥3mg/L;

Group4: AIP ≥ median & hs-CRP<1mg/L; Group5: AIP ≥ median & hs-CRP 1-3mg/L; Group6: AIP ≥ median & hs-CRP ≥3mg/L;

Table S5. Risk reclassification of cardiovascular disease based on AIP and hs-CRP.

|  | CVD | | Heart disease | | Stroke | |
| --- | --- | --- | --- | --- | --- | --- |
|  | HR (95% CI) | *P* value | HR (95% CI) | *P* value | HR (95% CI) | *P* value |
| Scenario1 (N of participants) |  |  |  |  |  |  |
| hs-CRP<1mg/L (N= 4343) |  |  |  |  |  |  |
| AIP < median | 1(Ref.) |  | 1(Ref.) |  | 1(Ref.) |  |
| AIP ≥ median | 1.272 (1.092, 1.482) | 0.002 | 1.106 (0.926, 1.322) | 0.266 | 1.603 (1.237, 2.077) | <0.001 |
| hs-CRP ≥ 1 mg/L(N=4420) |  |  |  |  |  |  |
| AIP < median | 1(Ref.) |  | 1(Ref.) |  | 1(Ref.) |  |
| AIP ≥ median | 1.411 (1.233, 1.614) | <0.001 | 1.297 (1.105, 1.523) | 0.001 | 1.539 (1.245, 1.902) | <0.001 |
| Scenario2 |  |  |  |  |  |  |
| AIP < median (N=4380) |  |  |  |  |  |  |
| hs-CRP < 1 mg/L | 1(Ref.) |  | 1(Ref.) |  | 1(Ref.) |  |
| hs-CRP ≥ 1 mg/L | 1.114 (0.953, 1.302) | 0.175 | 1.010 (0.842, 1.211) | 0.917 | 1.394 (1.074, 1.809) | 0.012 |
| AIP ≥ median(N=4383) |  |  |  |  |  |  |
| hs-CRP < 1 mg/L | 1(Ref.) |  | 1(Ref.) |  | 1(Ref.) |  |
| hs-CRP ≥ 1 mg/L | 1.219 (1.070, 1.388) | 0.003 | 1.167 (0.999, 1.363) | 0.052 | 1.331 (1.079, 1.641) | 0.007 |

Scenario 1: Effect of AIP on cardiovascular diseases between hs-CRP groups; Scenario 2: Effect of hs-CRP on cardiovascular diseases between AIP groups. HR, hazard ratio; CI, confidence interval; AIP: Atherogenic Index of Plasma; hs-CRP: high-sensitivity C-reactive protein; CVD: cardiovascular disease. Model was adjusted for age, sex, residence, marriage, education, smoking and drinking, Frailty and household fuel use, BMI level, hypertension, diabetes, Arthritis or Rheumatism, and history of medication use for hypertension, diabetes, and dyslipidemia.

Table S6: Sensitivity analyses of co-exposure for AIP and hs-CRP on CVD events.

|  | Sensitivity analysis 1 (n=6881) | | Sensitivity analysis 2 (n=2923) | |
| --- | --- | --- | --- | --- |
|  | HR (95% CI) | *P* value | HR (95% CI) | *P* value |
| Heart disease |  |  |  |  |
| Group1 | 1(Ref.) |  | 1(Ref.) |  |
| Group2 | 1.021(0.838, 1.244) | 0.839 | 1.100(0.810, 1.495) | 0.541 |
| Group3 | 1.135(0.936, 1.375) | 0.197 | 1.271(0.947, 1.706) | 0.110 |
| Group4 | 1.219(1.008, 1.473) | 0.041 | 1.453(1.107, 1.907) | 0.007 |
| Stroke |  |  |  |  |
| Group1 | 1(Ref.) |  | 1(Ref.) |  |
| Group2 | 1.472(1.116, 1.941) | 0.006 | 1.492(0.945, 2.353) | 0.086 |
| Group3 | 1.464(1.105, 1.940) | 0.008 | 1.617(1.027, 2.544) | 0.038 |
| Group4 | 1.652(1.271, 2.149) | < 0.001 | 1.949(1.279, 2.970) | 0.002 |
| CVD |  |  |  |  |
| Group1 | 1(Ref.) |  | 1(Ref.) |  |
| Group2 | 1.143(0.966, 1.352) | 0.120 | 1.240(0.951, 1.618) | 0.112 |
| Group3 | 1.262(1.070, 1.489) | 0.006 | 1.413(1.091, 1.830) | 0.009 |
| Group4 | 1.330(1.136, 1.556) | < 0.001 | 1.617(1.252, 2.087) | <0.001 |

|  | Sensitivity analysis 3 (n=7082) | | Sensitivity analysis 4 (n=6015) | |
| --- | --- | --- | --- | --- |
|  | HR (95% CI) | *P* value | HR (95% CI) | *P* value |
| Heart disease |  |  |  |  |
| Group1 | 1(Ref.) |  | 1(Ref.) |  |
| Group2 | 0.993(0.810, 1.217) | 0.946 | 1.03 (0.821-1.293) | 0.541 |
| Group3 | 1.131 (0.926, 1.382) | 0.226 | 1.144 (0.918-1.426) | 0.110 |
| Group4 | 1.228 (1.018, 1.481) | 0.032 | 1.391 (1.139-1.698) | 0.001 |
| Stroke |  |  |  |  |
| Group1 | 1(Ref.) |  | 1(Ref.) |  |
| Group2 | 1.534 (1.141, 2.062) | 0.005 | 1.58 (1.125-2.219) | 0.008 |
| Group3 | 1.509 (1.107, 2.057) | 0.009 | 2.067 (1.485-2.876) | <0.001 |
| Group4 | 1.821 (1.365, 2.429) | <0.001 | 2.044 (1.489-2.807) | <0.001 |
| CVD |  |  |  |  |
| Group1 |  |  | 1(Ref.) |  |
| Group2 | 1.079 (0.907, 1.285) | 0.391 | 1.149 (0.945-1.398) | 0.165 |
| Group3 | 1.209 (1.016, 1.440) | 0.032 | 1.39 (1.150-1.681) | <0.001 |
| Group4 | 1.351 (1.145, 1.593) | <0.001 | 1.457 (1.217-1.743) | <0.001 |

Model was adjusted for age, sex, residence, marriage, education, smoking, alcohol consumption, frailty and household fuel use, BMI level, hypertension, diabetes, Arthritis or Rheumatism, and history of medication use for hypertension, diabetes, and dyslipidemia. Sensitivity analysis1 was performed among 6881 participants without any missing data (except for physical activity); Sensitivity analysis 2 was conducted among 2,923 participants without any missing data, with additional adjustment for physical activity in the model; Sensitivity Analysis 3 was performed by excluding participants with a baseline history of medication use for hypertension, diabetes, and dyslipidemia; Sensitivity Analysis 4 was performed by excluding participants with Arthritis or Rheumatism.

Table S7: Comparison of models adjusted for waist circumference versus BMI in relation to CVD events.

|  | BMI | | waist circumference | |
| --- | --- | --- | --- | --- |
|  | HR (95% CI) | *P* value | HR (95% CI) | *P* value |
| Heart disease |  |  |  |  |
| Group1 | 1(Ref.) |  | 1(Ref.) |  |
| Group2 | 1.047(0.882, 1.242) | 0.602 | 1.088(0.904, 1.310) | 0.372 |
| Group3 | 1.173(0.992, 1.386) | 0.062 | 1.271(0.947, 1.706) | 0.050 |
| Group4 | 1.328(1.139, 1.548) | <0.001 | 1.453(1.107, 1.907) | 0.002 |
| Stroke |  |  |  |  |
| Group1 | 1(Ref.) |  | 1(Ref.) |  |
| Group2 | 1.399(1.091, 1.795) | 0.008 | 1.455(1.116, 1.897) | 0.006 |
| Group3 | 1.586(1.238, 2.032) | <0.001 | 1.500(1.145, 1.967) | 0.003 |
| Group4 | 2.021(1.615, 2.529) | <0.001 | 1.809(1.412, 2.317) | <0.001 |
| CVD |  |  |  |  |
| Group1 | 1(Ref.) |  | 1(Ref.) |  |
| Group2 | 1.140(0.983, 1.321) | 0.083 | 1.187(1.012, 1.392) | 0.035 |
| Group3 | 1.313(1.137, 1.517) | <0.001 | 1.319(1.126, 1.545) | <0.001 |
| Group4 | 1.546(1.355, 1.764) | < 0.001 | 1.497(1.293, 1.732) | <0.001 |

Abbreviations: hs-CRP: high-sensitivity C-reactive protein; AIP: Atherogenic Index of Plasma; CVD: cardiovascular disease; Group1: AIP<median & hs-CRP<1mg/L; Group2: AIP <median & hs-CRP≥1mg/L; Group3: AIP ≥ median & hs-CRP<1mg/L; Group4: AIP ≥ median & hsCRP≥1mg/L. Model 1 was adjusted for age, sex and BMI level; Model 2 was adjusted for age, sex and waist circumference.

Table S8: Stratified analyses of AIP and hs-CRP in relation to cardiovascular risk according to menopausal status in female participants.

|  | Premenopausal (n=1112) | | Postmenopausal (n=3226) | | *P* for interaction  (Group × Menopause) |
| --- | --- | --- | --- | --- | --- |
|  | HR (95% CI) | *P* value | HR (95% CI) | *P* value |  |
| Heart disease |  |  |  |  |  |
| Group1 | 1(Ref.) |  | 1(Ref.) |  |  |
| Group2 | 1.140(0.665, 1.953) | 0.637 | 1.056 (0.812, 1.374) | 0.682 | 0.650 |
| Group3 | 1.323 (0.785, 2.229) | 0.293 | 1.100 (0.845, 1.433) | 0.479 | 0.469 |
| Group4 | 1.841 (1.140, 2.974) | 0.013 | 1.219 (0.965, 1.540) | 0.097 | 0.096 |
| Stroke |  |  |  |  |  |
| Group1 | 1(Ref.) |  | 1(Ref.) |  |  |
| Group2 | 0.644 (0.203, 2.039) | 0.454 | 1.587 (1.067, 2.361) | 0.023 | 0.159 |
| Group3 | 1.183 (0.485, 2.885) | 0.712 | 1.569 (1.039, 2.369) | 0.032 | 0.390 |
| Group4 | 1.988 (0.934, 4.234) | 0.075 | 1.991 (1.388, 2.857) | <0.001 | 0.988 |
| CVD |  |  |  |  |  |
| Group1 | 1(Ref.) |  | 1(Ref.) |  |  |
| Group2 | 1.082 (0.633, 1.852) | 0.773 | 1.236 (0.986, 1.551) | 0.067 | 0.856 |
| Group3 | 1.447 (0.910, 2.303) | 0.119 | 1.200(0.950, 1.516) | 0.125 | 0.499 |
| Group4 | 1.913(1.241, 2.948) | < 0.001 | 1.412 (1.150, 1.732) | <0.001 | 0.160 |

Abbreviations: hs-CRP: high-sensitivity C-reactive protein; AIP: Atherogenic Index of Plasma; CVD: cardiovascular disease; Group1: AIP<median & hs-CRP<1mg/L; Group2: AIP <median & hs-CRP≥1mg/L; Group3: AIP ≥ median & hs-CRP<1mg/L; Group4: AIP ≥ median & hsCRP≥1mg/L. Model was adjusted for age, sex, residence, marriage, education, smoking, alcohol consumption, frailty and household fuel use, BMI level, hypertension, diabetes, Arthritis or Rheumatism, and history of medication use for hypertension, diabetes, and dyslipidemia.

Table S9**.** Multivariable models additionally adjusted for menopausal status in female participants.

|  | Model 1 | | Model 2 | |
| --- | --- | --- | --- | --- |
|  | HR (95% CI) | P value | HR (95% CI) | P value |
| Heart disease |  |  |  |  |
| Group1 | 1(Ref.) |  |  |  |
| Group2 | 1.120 (0.890, 1.409) | 0.333 | 1.059 (0.830, 1.351) | 0.644 |
| Group3 | 1.242 (0.995, 1.549) | 0.055 | 1.129 (0.891, 1.431) | 0.315 |
| Group4 | 1.417 (1.162, 1.730) | < 0.001 | 1.150 (0.917, 1.442) | 0.227 |
| Stroke |  |  |  |  |
| Group1 | 1(Ref.) |  |  |  |
| Group2 | 1.480 (1.030, 2.127) | 0.034 | 1.393 (0.954, 2.034) | 0.086 |
| Group3 | 1.610 (1.125, 2.305) | 0.009 | 1.438 (0.986, 2.098) | 0.059 |
| Group4 | 1.983 (1.443, 2.726) | < 0.001 | 1.622 (1.142, 2.303) | 0.007 |
| CVD |  |  |  |  |
| Group1 | 1(Ref.) |  |  |  |
| Group2 | 1.270 (1.039, 1.553) | 0.020 | 1.209 (0.978, 1.495) | 0.079 |
| Group3 | 1.353 (1.111, 1.647) | 0.003 | 1.234 (1.001, 1.522) | 0.049 |
| Group4 | 1.577 (1.322, 1.882) | < 0.001 | 1.281 (1.050, 1.563) | 0.015 |

Abbreviations: HR, hazard ratio; CI, confidence interval; BMI, body mass index; AIP: Atherogenic Index of Plasma; hs-CRP: high-sensitivity C-reactive protein; CVD: cardiovascular disease; Group1: AIP<median & hs-CRP<1mg/L; Group2: AIP <median & hs-CRP≥1mg/L; Group3: AIP ≥ median & hs-CRP<1mg/L; Group4: AIP ≥ median & hs-CRP≥1mg/L. Model 1: adjusted for age, residence, marriage, education and menopausal status; Model 2: adjusted for age, residence, marriage, education, smoking, alcohol drinking, frailty and household fuel use, BMI level, hypertension, diabetes, Arthritis or Rheumatism, menopausal status, history of medication use for hypertension, diabetes, and dyslipidemia.

Table S10: Sensitivity analyses of co-exposure for AIP and other inflammatory biomarkers on CVD events.

|  | WBC | | PLT | | PWR | |
| --- | --- | --- | --- | --- | --- | --- |
|  | HR (95% CI) | *P* value | HR (95% CI) | *P* value | HR (95% CI) | *P* value |
| Heart disease |  |  |  |  |  |  |
| Group1 | 1(Ref.) |  | 1(Ref.) |  | 1(Ref.) |  |
| Group2 | 1.007 (0.841, 1.205) | 0.943 | 0.959 (0.801, 1.148) | 0.649 | 1.008 (0.842, 1.207) | 0.932 |
| Group3 | 1.237 (1.041, 1.470) | 0.016 | 1.188 (1.000, 1.411) | 0.050 | 1.268 (1.067, 1.506) | 0.007 |
| Group4 | 1.286 (1.092, 1.515) | 0.003 | 1.278 (1.082, 1.510) | 0.004 | 1.262 (1.060, 1.504) | 0.009 |
| Stroke |  |  |  |  |  |  |
| Group1 | 1(Ref.) |  | 1(Ref.) |  | 1(Ref.) |  |
| Group2 | 1.212 (0.936, 1.569) | 0.146 | 0.974 (0.751, 1.262) | 0.840 | 0.994(0.767, 1.288) | 0.962 |
| Group3 | 1.903 (1.494, 2.423) | <0.001 | 1.464 (1.154, 1.857) | 0.002 | 1.592 (1.259, 2.013) | <0.001 |
| Group4 | 1.909 (1.514, 2.406) | <0.001 | 1.964 (1.568, 2.460) | <0.001 | 1.892 (1.497, 2.392) | <0.001 |
| CVD |  |  |  |  |  |  |
| Group1 | 1(Ref.) |  | 1(Ref.) |  | 1(Ref.) |  |
| Group2 | 1.014 (0.869, 1.185) | 0.857 | 0.950 (0.813, 1.109) | 0.517 | 1.037 (0.888, 1.211) | 0.646 |
| Group3 | 1.413 (1.221, 1.635) | <0.001 | 1.288 (1.113, 1.491) | <0.001 | 1.423 (1.229, 1.646) | <0.001 |
| Group4 | 1.472 (1.281, 1.691) | <0.001 | 1.509 (1.311, 1.735) | <0.001 | 1.507 (1.300, 1.747) | <0.001 |

Abbreviations: hs-CRP: high-sensitivity C-reactive protein; AIP: Atherogenic Index of Plasma; CVD: cardiovascular disease; PWR, platelet to white blood cell ratio;

Group1: AIP<median & WBC (PLT or PWR) < median; Group2: AIP <median & WBC (PLT or PWR) ≥ median; Group3: AIP ≥ median &WBC (PLT or PWR) < median; Group4: AIP ≥ median &WBC (PLT or PWR) ≥ median. Model was adjusted for age, sex, residence, marriage, education, smoking, alcohol consumption, frailty and household fuel use, BMI level, hypertension, diabetes, Arthritis or Rheumatism, and history of medication use for hypertension, diabetes, and dyslipidemia.

**Figure S1**: Cumulative Hazard of CVD by AIP and hs-CRP level.


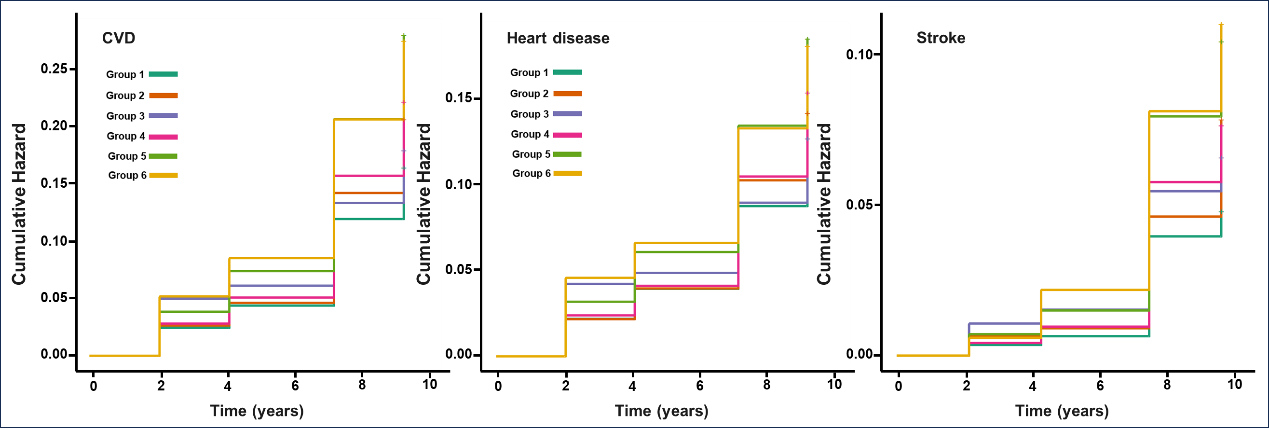


Abbreviations: CVD: cardiovascular disease; AIP, atherogenic index of plasma, hs-CRP, high-sensitivity C-reactive protein; median of AIP: -0.088. Group1: AIP<median & hs-CRP<1mg/L; Group2: AIP <median & hs-CRP 1-3 mg/L; Group3: AIP <median & hs-CRP≥3mg/L;Group4: AIP ≥ median & hs-CRP<1mg/L; Group5: AIP ≥ median & hs-CRP 1-3mg/L; Group6: AIP ≥ median & hs-CRP ≥3mg/L.

Figure S2. Mutual mediation effects of AIP and hs-CRP on heart disease.


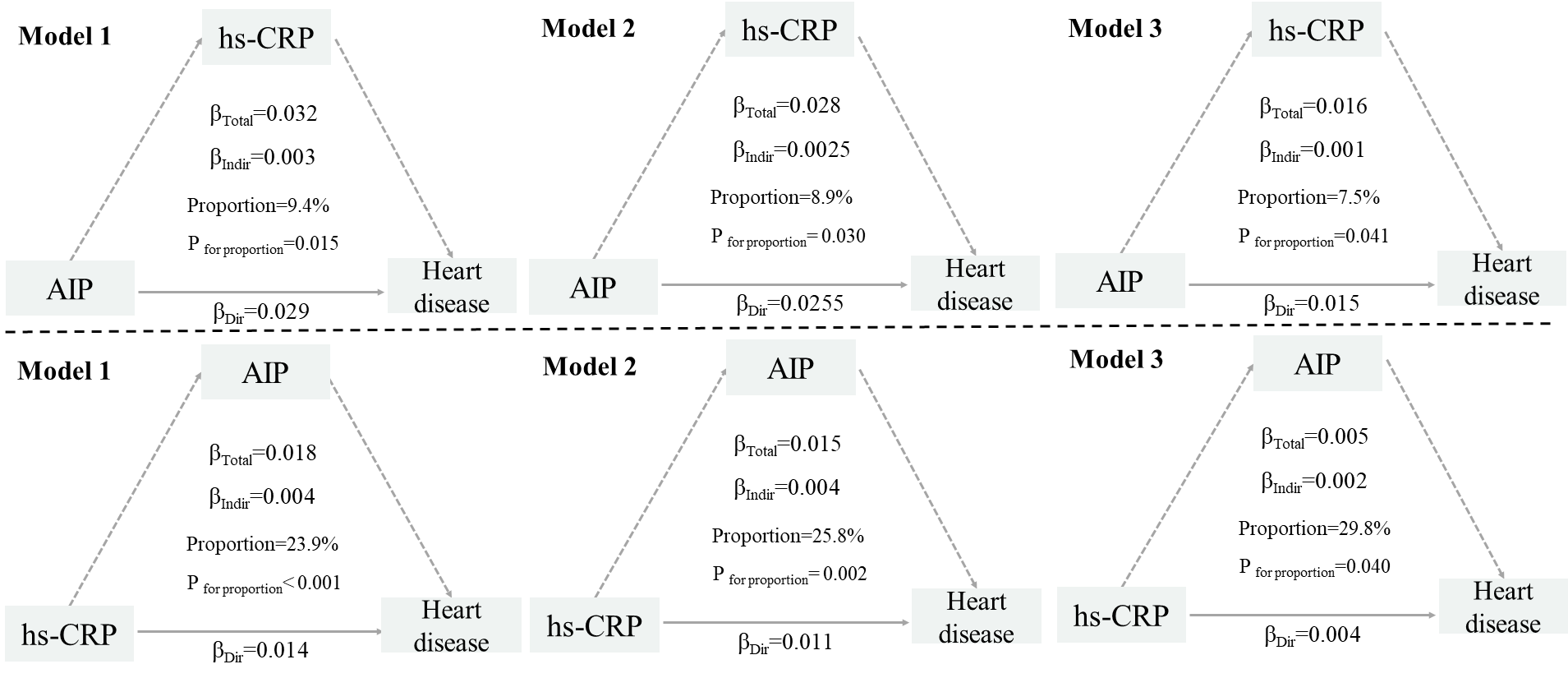


AIP: Atherogenic Index of Plasma; hs-CRP: high-sensitivity C-reactive protein.

Model 1: adjusted for age, sex, residence, marriage, education;

Model 2: model 1 plus smoking, alcohol consumption, Frailty and household fuel use;

Model 3: model 2 plus BMI level, hypertension, diabetes, Arthritis or Rheumatism, and history of medication use for hypertension, diabetes, and dyslipidemia.

Figure S3. Mutual mediation effects of AIP and hs-CRP on stroke.


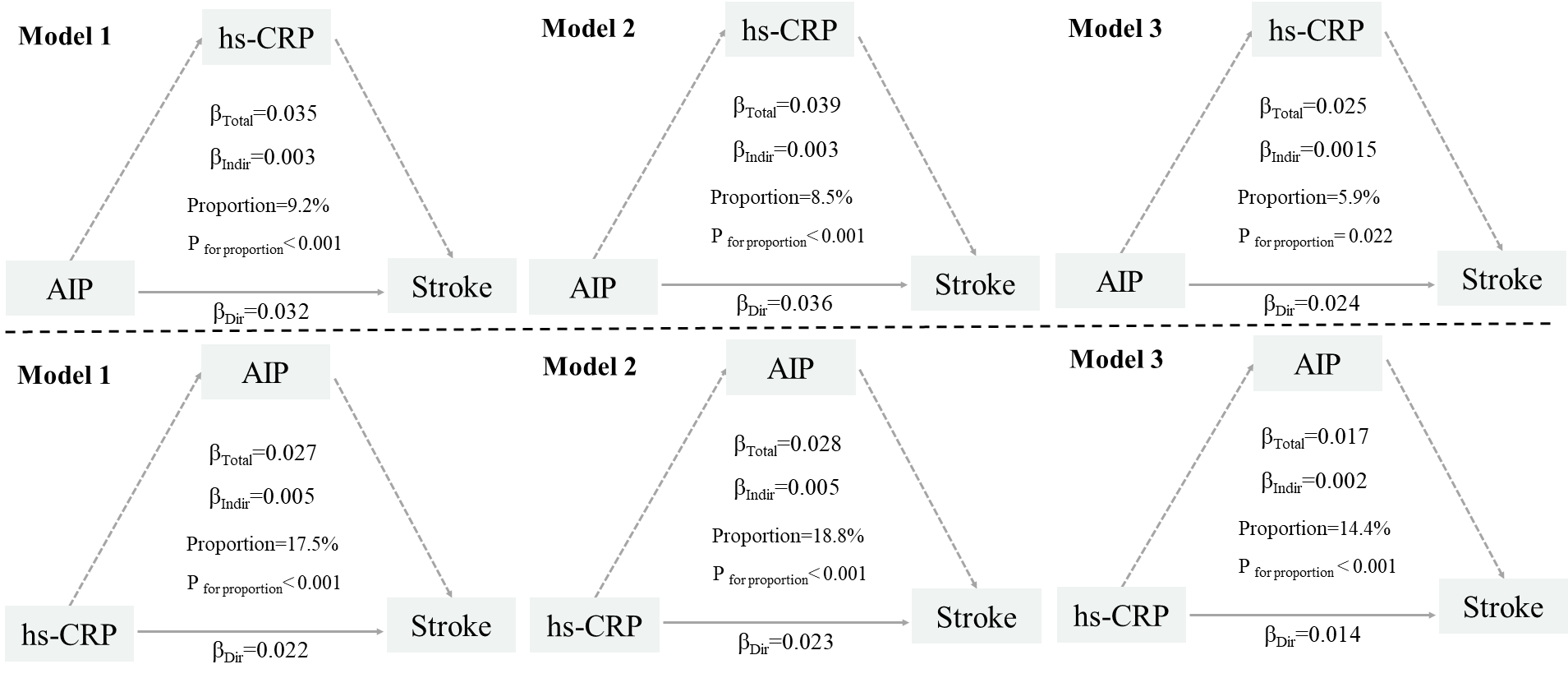


Figure S5. Mutual mediation effects of the AIP and hs-CRP on stroke. AIP: Atherogenic Index of Plasma; hs-CRP: high-sensitivity C-reactive protein.

Model 1: adjusted for age, sex, residence, marriage, education;

Model 2: model 1 plus smoking, alcohol consumption, frailty and household fuel use;

Model 3: model 2 plus BMI level, hypertension, diabetes, Arthritis or Rheumatism, and history of medication use for hypertension, diabetes, and dyslipidemia.

Figure S4. ROC curves for AIP, hs-CRP, and their combination in predicting CVD stratified by age groups.


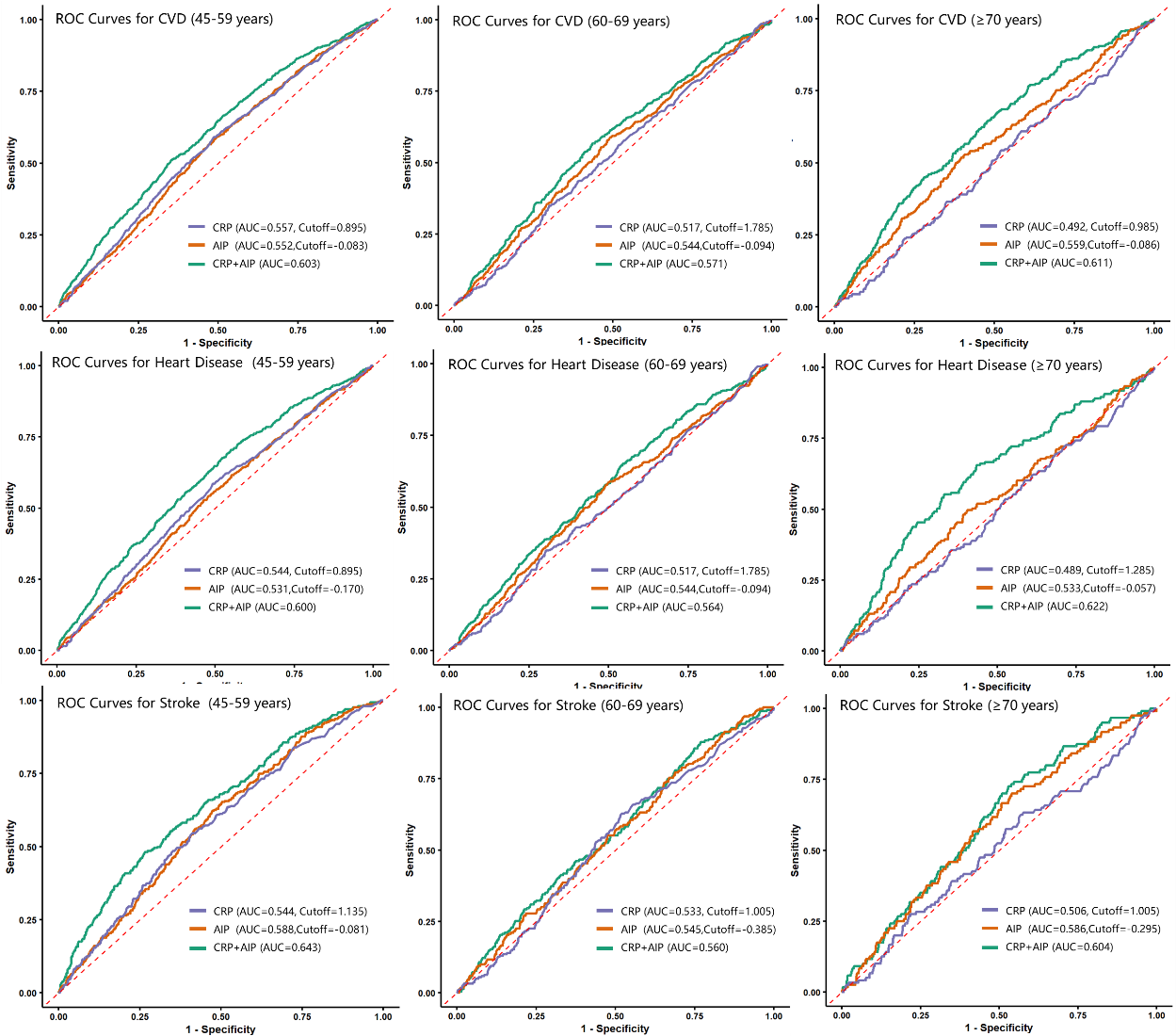


Figure S5. ROC curves for AIP, hs-CRP, and their combination in predicting CVD stratified by BMI levels.


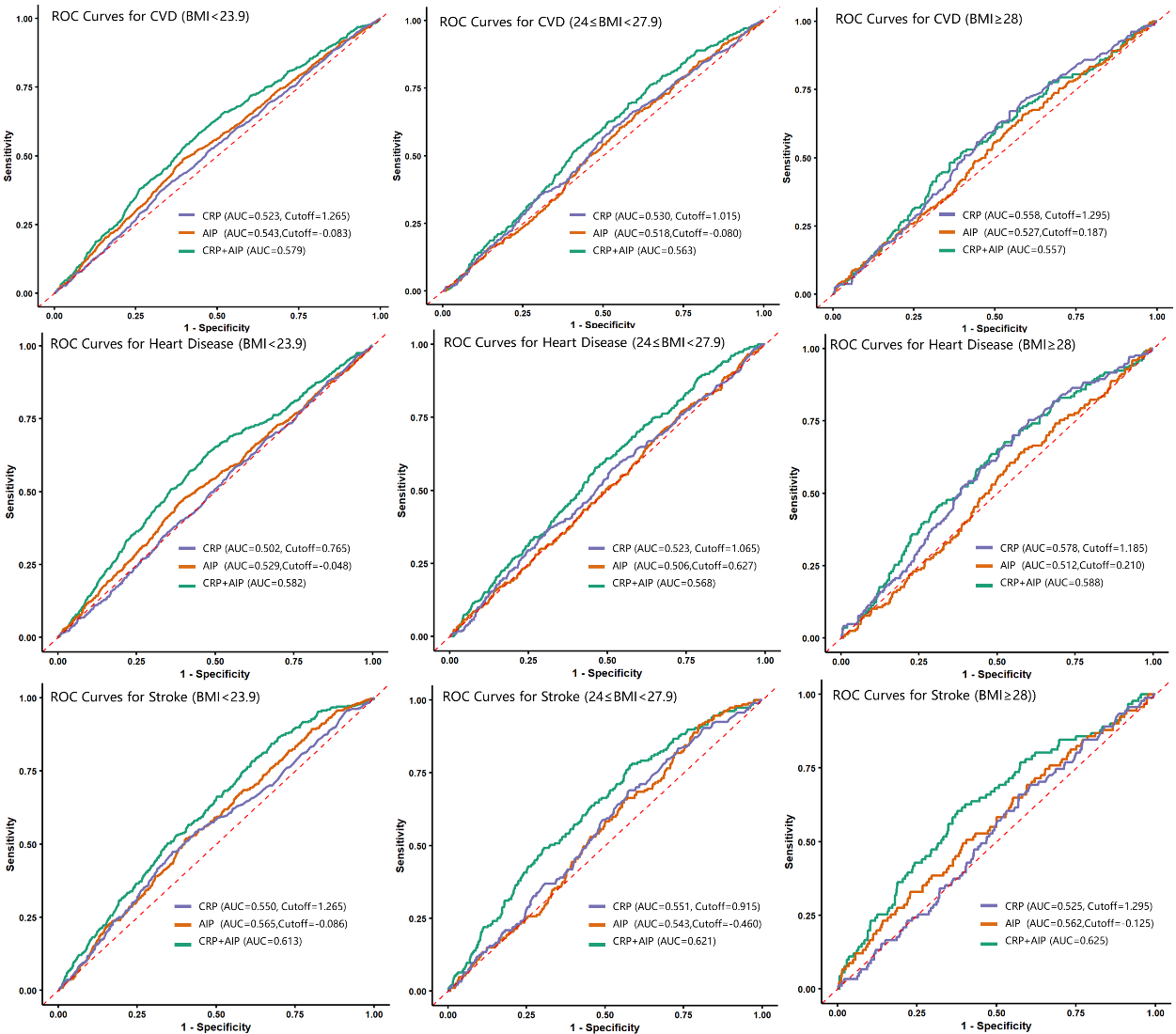


Figure S6. Distribution Characteristics of AIP and hs-CRP.


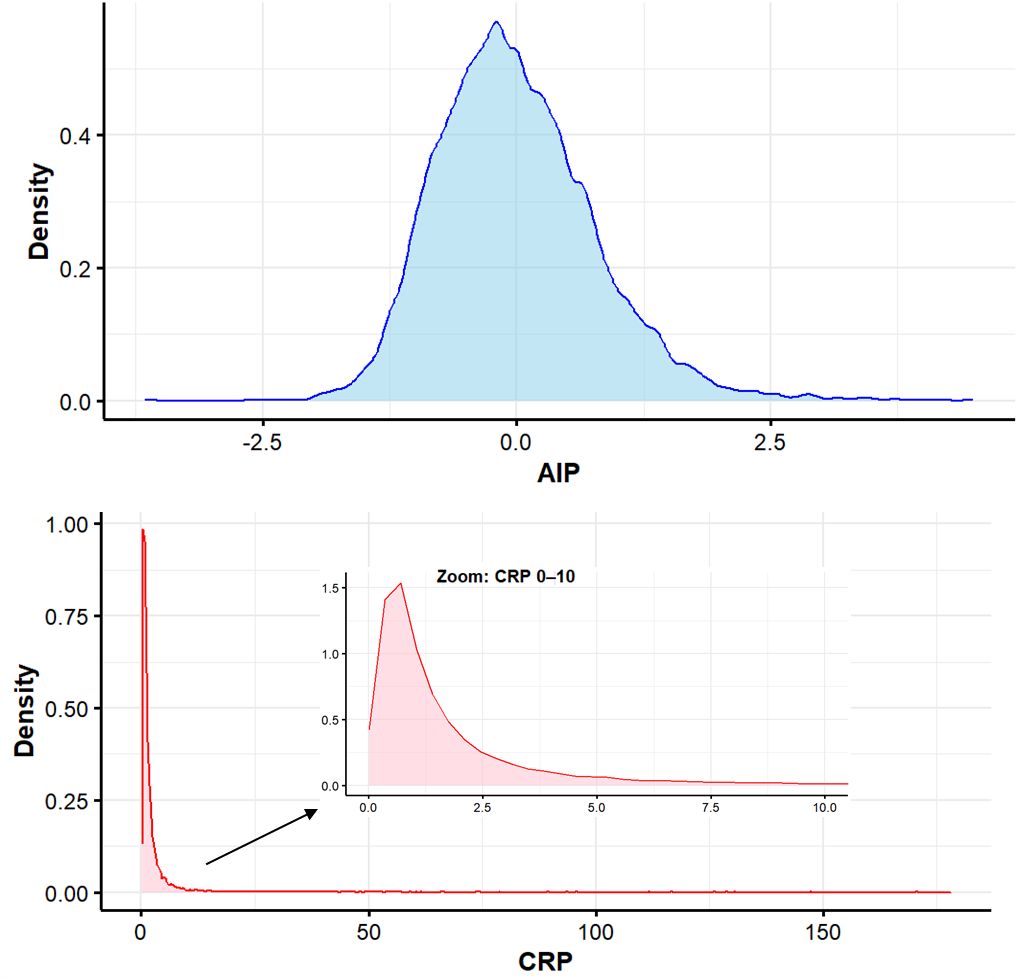

Supplement: Supplementary file 1 [file DataSheet1.docx]
